# Substances and substance combinations among accidental substance-related acute toxicity deaths (AATDs) in Canada from 2016 to 2017

**DOI:** 10.1186/s12889-025-22777-2

**Published:** 2025-12-03

**Authors:** Raahyma Ahmad, Tanya Kakkar, Jenny Rotondo, Keltie Hamilton, Matthew J. Bowes, Graham Jones, Cindy Leung Soo, Amanda VanSteelandt

**Affiliations:** 1 Substance-Related Harms Division, Public Health Agency of Canada, 785 Carling Ave, Ottawa, ON K1A 0K9 Canada; 2 Nova Scotia Medical Examiner Service, Department of Justice, Halifax, NS Canada; 3 Department of Laboratory Medicine and Pathology, Faculty of Medicine and Dentistry, University of Alberta, Edmonton, AB Canada; 4 Centre for Emergency Preparedness, Public Health Agency of Canada, Ottawa, ON Canada

**Keywords:** Acute toxicity, Canada, Chart review study, Coroner, Death investigations, Drug overdose, Medical examiner, Mortality, Poisoning, Polydrug, Polysubstance, Mixed-drug toxicity, Opioids

## Abstract

**Background:**

Canada has seen a rise in substance-related accidental acute toxicity deaths (AATDs) in recent years. Research indicates that fentanyl opioids, non-fentanyl opioids, and stimulants are classes of concern and that multidrug AATDs have increased. However, there is limited information regarding the specific substances involved. This study aims to identify the substances and substance combinations as well as substance classes and substance class combinations most often involved in AATDs across Canada between 2016 and 2017. It also examines variations in substances by year and across sociodemographic, socioeconomic, and geographic factors.

**Methods:**

Data were abstracted from the coroner and medical examiner files of all AATDs that occurred across Canada between 2016 and 2017. Top substances and classes detected in or contributing to AATDs were identified based on toxicology reports and cause of death statements. AATDs were stratified by year of death, age, sex, residence community type, neighbourhood income quintile, and province/region to understand variations in the substances contributing to AATDs. Combinations of substances and classes contributing to death were examined with UpSet plots and trends of select substances were visualized over time with ribbon charts. An algorithm was developed to report the source and origin of the substances based on prescription history and scene evidence.

**Results:**

Fentanyl, cocaine, alcohol, and methamphetamine were the top substances contributing to the 7,902 AATDs identified between 2016 and 2017 in Canada. While stimulants and opioids were the most common substance classes contributing to AATDs, other classes, including benzodiazepines and acetaminophen also emerged as classes among the top contributors. Between 2016 and 2017, the proportion of AATDs attributable to diacetylmorphine (heroin) per quarter decreased while the proportion of AATDs attributable to carfentanil per quarter increased. AATDs involving more than one substance occurred across all sociodemographic, socioeconomic, and geographic groups. Substances contributing to AATDs more commonly originated from non-pharmaceutical sources than from pharmaceutical sources.

**Conclusions and impacts:**

Specific substances and substance combinations contributing to deaths vary over time and geographic areas. Opioids and stimulants are both detected in and contribute to a majority of AATDs, but the substance-related acute toxicity death crisis is complex and attributable to many substance classes. Understanding these differences will allow for targeted substance-related policies, prevention, and harm reduction efforts.

**Supplementary Information:**

The online version contains supplementary material available at 10.1186/s12889-025-22777-2.

## Background

Substance-related acute toxicity deaths (also referred to as poisoning or overdose deaths) have been on the rise in Canada since the early 2000s. While the overdose crisis continues to evolve today, the years 2016 and 2017 represent a significant period in terms of shifts in drug supply and substances contributing to these deaths [1–4]. In 2017, there were 3,924 opioid-related deaths in Canada, which was a 39% increase from 2016 [3]. While the majority of these deaths involved fentanyl and other opioids, most opioid-related deaths also involved non-opioid substances such as benzodiazepines, stimulants, and alcohol, reflecting an increase in multidrug toxicity deaths in Canada [4–8].

Multidrug (or polysubstance) use refers to the concurrent intentional or unintentional use of multiple substances. Multidrug use can increase the risk of acute toxicity, as the combination of substances can have unpredictable and additive or synergistic pharmacological effects [9]. Multiple substances can be used to intensify the effects of substance use after a buildup of tolerance or to achieve a desired effect that cannot be achieved with a single substance alone [10, 11]. Multidrug use is often associated with certain risk factors, such as unaddressed physical or mental health needs, chronic pain and a history of substance use disorders. These factors can increase the likelihood of using multiple substances and make it more difficult for individuals to seek and receive appropriate treatment [12, 13].

Despite the increasing concern around accidental substance-related acute toxicity deaths (AATDs) in Canada, there is limited information on the specific substances involved in these deaths. Published data often only report on the substance classes involved or use International Classification of Death (ICD) codes, which group similar substances and do not specify all specific substances contributing to death [7]. Additionally, there is limited information on deaths that were not opioid related in 2016 and 2017 [3]. Recent studies indicate that substances beyond opioids may have been contributing to the overdose crisis since surveillance of opioid-related harms began in Canada in 2016 [3, 5]. More detailed information is essential to understanding how new substances emerge and displace others and the risks of different substance combinations. In-depth national-level analyses exploring the substances and substance combinations that contribute to deaths can address knowledge gaps by providing a more complete picture of substance-related acute toxicity deaths, including the extent of multidrug toxicity.

To prevent acute toxicity deaths, it is also important to understand the source and origin of the substances that contribute to them. Substances may be pharmaceutical or non-pharmaceutical in origin based on where they were manufactured. They can also have multiple sources based on how they were accessed; personal prescriptions are those prescribed directly to the individual for their own use while diverted prescriptions are prescribed medications that are used by individuals who were not prescribed the medication. The well documented association between high dose opioid prescribing and opioid-related acute toxicity events prompted changes in the formulation of pharmaceutical opioids and rates of opioid prescribing over time in Canada [14]. Studies examining the impact of these changes on the role of prescribed versus non-prescribed opioids in fatal acute toxicity events revealed a decreasing contribution of prescribed opioids compared to non-prescribed fentanyl and related analogues [15, 16]. However, these analyses took place at the sub-national level. The use of diverted prescriptions is also a key factor in understanding the role of the pharmaceutical supply overall, however national-level data are not currently available to explore this. The dynamics between use of substances of pharmaceutical (personal or diverted prescriptions) and non-pharmaceutical origin have also been shown to vary by sex, however differences by other sociodemographic, socioeconomic, and geographical characteristics warrant greater attention [15, 16].

Provincial and territorial coroners and medical examiners investigate deaths that are unexpected, unexplained or that occur by violence, including acute toxicity deaths. Their records, which include documents such as death certificates, toxicology reports and medical history are a rich source of information about the acute toxicity death problem in Canada. Toxicology data and cause of death statements offer enhanced granularity by identifying specific substances contributing to death. This detailed information allows for accurate classification of substances contributing to deaths, as each substance is recognized individually rather than grouped into broader substance classes. Consequently, this enables more accurate identification of multidrug deaths, particularly when multiple substances within the same class are involved [7, 8, 17, 18]. Death investigation files also often include prescription histories and other information permitting the determination of the source and origin of substances detected on toxicology.

Overall, gaps exist in our understanding of the substances, substance combinations, and sources and origin of substances involved in AATDs in Canada. This study aims to address these gaps by using a retrospective chart review of coroner and medical examiner files on accidental acute toxicity deaths (AATDs) across Canada in 2016 and 2017 to identify specific substances and substance combinations that were commonly detected or contributed to death, their source and origin, and differences in substance involvement across time by sociodemographic, socioeconomic, and geographic characteristics [19]. Findings should improve our understanding of the substance landscape at the beginning of the overdose crisis in Canada and how it has since evolved.

## Methods

### Data collection

Data on AATDs were obtained from a retrospective, population-based, cross-sectional chart review of coroner and medical examiner files on substance-related acute toxicity deaths that occurred in Canada between 2016 and 2017. Acute toxicity was defined as the direct effects of the administration of exogenous substances, where one or more of the substances was a drug or alcohol. Deaths due solely to chronic substance use or associated with medical assistance in dying, palliative or comfort care, homicide, occupational exposure, trauma where an intoxicant contributed to the circumstances of the injury (such as a motor vehicle accident), adverse drug effects (such as anaphylactic shock), or products of combustion (such as carbon monoxide) were excluded. Coroner and medical examiner files in all provinces and territories pertaining to the deaths of people who met the study case definition were abstracted to collect information on sociodemographic factors, drug and medical history, circumstances of death, and toxicological findings using a standardized data collection tool [19]. British Columbia data were only available for people who experienced AATDs involving unregulated drugs and/or drugs sold illicitly. As such, British Columbia data for people who experienced AATDs due solely to prescribed substances or alcohol were not available. A detailed explanation of the chart review design and abstraction process has been reported elsewhere [19].

In Canada, death investigations fall under the jurisdiction of provinces and territories which have developed their own coroner or medical examiner systems to investigate deaths that are unexpected, unexplained, or a result of injuries or drugs [20]. Coroners and medical examiners investigate deaths to determine the identity of the deceased and the cause and manner of death, which are recorded on the death certificate. They collect a variety of information during the course of their investigation, including the circumstances of death, medical history, evidence from the scene of death, and witness statements. Substances detected were identified from detailed substance-level toxicology reports of the person who died or the medical files for those who died after extended hospital stays. Substances contributing to death were identified from both the toxicology report and explicit cause of death statements from coroners and medical examiners, who considered factors such as quantities detected, dose tolerance, and medical history when determining the cause of death [21]. Deaths where substances contributing to death were determined from cause of death statements may lack toxicology data due to factors such as body decomposition, prolonged hospital stays, or missing records in the death investigation file. As a result, these cases may only have information on substances identified as contributing to death, and may lack data on substances detected. For British Columbia, all substances listed in the electronically available files were substances detected that were deemed relevant to death and therefore were listed as both detected and contributing to death. Some substances identified in toxicology reports were clearly metabolites of single substances and were therefore used to impute presence of the parent substance and subsequently excluded from the study (for example, as the presence of norbuprenorphine was used as evidence for the presence of buprenorphine, norbuprenorphine was excluded from the list of substances involved in AATDs). Substances that contribute to death independently and were possible metabolites of other substances were maintained in the list of substances involved. Substances were categorized into substance classes [see Additional File 1], and variables were derived for substance classes that were detected and that contributed to death for each person who died of acute toxicity.

Substance origin was classified as pharmaceutical for substances manufactured in a pharmaceutical setting and non-pharmaceutical for substances not intended for human medical use (such as non-pharmaceutical inhalants, industrial or household chemicals, or veterinary drugs), food compounds, and illegal substances. For substances manufactured in a pharmaceutical setting, the source was ‘prescribed’ when there was evidence in the coroner or medical examiner file indicating that the substance was prescribed to the individual and ‘diverted’ when there was evidence indicating that the substance was prescribed to someone other than the person who died. In this study, ‘evidence’ refers to substance information documented during the death investigation processes, including documented prescription records for the person who died obtained through healthcare provider interviews or prescription database linkages. It also includes presence of prescription drugs found at the scene, such as prescription bottles labeled with the person's name or another individual’s name. Additionally, reports from witnesses or family members may provide information suggesting a pharmaceutical origin. Prescription histories were collected for any medication that was prescribed up to 6 months before death if there was access to pharmacy records for this information or if there was information on specific dates for each prescription. If information on prescribed medication was available but it was unclear whether the medication was prescribed within the last six months, it was still documented. The absence of evidence indicates that none of these specific sources of information were available as evidence to determine that the origin was pharmaceutical. The most appropriate origin classifications for substances that could be of either pharmaceutical origin, non-pharmaceutical origin, or both were determined based on an algorithm which considered additional study evidence such as history of substance use and other substances detected in toxicology [see Additional File 2].

A substance may have more than one origin and/or have been acquired from more than one source, therefore origin categories (pharmaceutical versus non-pharmaceutical) and pharmaceutical source categories (prescribed versus diverted) for a particular substance may not sum to 100%. Substance origin and source data were not available for British Columbia nor for a subset of records from Ontario that were only available electronically; however, certain substances that are known to be exclusively of pharmaceutical or non-pharmaceutical origin were assigned an origin. Due to lack of information on prescription history, substances that could be of either origin such as fentanyl, codeine, and morphine were assigned an “unknown” origin for British Columbia deaths. Information on origin for these substances were imputed, where possible, for the electronic records from Ontario based on the algorithm and available prescription history data.

### Data analysis

Substances detected or contributing to death in at least 5% of all AATDs were identified and the proportion of AATDs in which these substances were detected, contributing to death when detected, and contributing to death alone were calculated. The proportion of deaths where a substance ‘contributed to death when detected’ was calculated by first identifying the total number of deaths in which the substance was detected. Then, among those deaths, the number where the substance contributed to death was used as the numerator, and the total number of deaths where it was detected served as the denominator. The proportion of deaths where a substance ‘contributed to death alone’ was determined by dividing the number of deaths in which the substance was the sole contributor by the total number of deaths where it contributed to death. The source and origin of each substance was also determined. A complete list of all substances contributing to death can be found in Additional file 3.

To examine trends by residence community type (urbanicity) and socioeconomic status, substances detected in and contributing to at least 10% of AATDs were identified among people who lived in urban or rural communities, and for each neighbourhood income quintile. Neighbourhood income quintiles (variable QAATIPPE) are derived based on income after-tax at the dissemination area (DA) level and were constructed separately for census metropolitan areas (CMAs), census agglomerations (CAs), and residual areas (DAs outside of CMA/CAs) [22, 23]. For the purposes of this study, urban municipalities included census metropolitan areas with populations of at least 100,000 residents and census agglomerations with populations of at least 10,000 residents. Rural municipalities were defined as all areas outside of census metropolitan areas and agglomerations. Data on census metropolitan areas, census agglomerations, and neighbourhood income quintiles were acquired through linkage of the study dataset to the Statistics Canada Postal Code Conversion File Plus using the postal code of residence for the person who died and detailed information on these methods are available elsewhere [22, 24]. Those with unknown postal codes were characterized as having an unknown municipality of residence. A sensitivity analysis was conducted for people who belonged to the unknown income quintile and unknown community type, and notably, 25% and 60% respectively were unhoused at the time of their death.

To examine trends in substances contributing to AATDs over time, R Studio version 1.4.1106 was used to generate standardised control p-charts (proportion charts) using the Qicharts package [25]. Points were plotted in standard deviation units along with a centre line at zero and control limits at 3 and -3 to identify substances with significant changes over the two-year study period. The centre line was based on the weighted mean of the proportion of AATDs that each substance contributed to per quarter during the first three quarters (January to September) of 2016, to establish a baseline for each substance during the study period. The third quarter of 2016 was used as the cutoff period for the baseline as it marked the declaration of the opioid crisis within Canada [5, 26]. For each substance, the control p-chart plotted the proportion of AATDs that substance contributed to per quarter (January 2016 to March 2016, April 2016 to June 2016, etc.). This allowed us to determine which substances contributed to a significantly higher or lower proportion of AATDs per quarter after the opioid crisis was first declared in Canada in April of 2016 [26]. Substances that contributed to AATDs more or less often per quarter than expected based on the control limits after the first three quarters of 2016 and contributed to more than 10 deaths over the two-year study period were visualized in ribbon charts. Two ribbon charts were generated; the first chart was specific to non-opioids and the second was specific to opioids.

To explore differences between people who died of multidrug toxicity and single-drug toxicity, proportions of people in each group with a given sociodemographic or area-level characteristic were calculated. Multidrug toxicity deaths were defined as deaths where more than one substance contributed to the death or where multiple-drug toxicity was mentioned as the cause of death in the coroner or medical examiner statement.

Using R Studio version 1.4.1106, the package ComplexUpset was utilized to generate UpSet plots showing the top 20 substances or substance combinations contributing to deaths by year, with one set demonstrating these trends at the substance specific level and the other set at the substance class level [27, 28]. The number of AATDs for each substance or substance combination by sex were shown with coloured bar graphs in both sets of UpSet plots. Tripsit wiki’s Guide to Drug Combinations Chart was used to colour code substance combinations as dangerous, unsafe, or requiring caution in the substance specific UpSet plot [29]. Tripsit’s drug combination chart is a widely used, quick and free reference guide used for harm reduction, with a statement cautioning users to conduct additional research before making decisions. Combinations classified as “dangerous” are those that are extremely harmful and should not be taken together as they can result in severe health complications and may even be fatal. Combinations classified as “unsafe” should be avoided as they can result in physical bodily harm. “Caution” combinations are those that should be taken with considerable caution as they may not necessarily cause physical harm but can have undesirable effects on the body, such as physical unease and overstimulation, and overuse and additive effects of this combination can be hard to anticipate and can possibly result in health problems [29]. The top substances and substance combination trends were identified for provinces/regions. Manitoba and Saskatchewan were grouped together as the Prairie region, Nova Scotia, Newfoundland and Labrador, Prince Edward Island and New Brunswick were grouped as the Atlantic region and Nunavut, Yukon and the Northwest Territories were grouped together as the Northern region due to small numbers in these provinces/territories. British Columbia, Alberta, Ontario, and Quebec were not grouped with any other provinces/territories.

Descriptive analyses of substances contributing to death that were of pharmaceutical origin, with a prescribed, diverted or unknown source, and non-pharmaceutical origin were conducted by sex, age group, year of death, neighbourhood income quintile, urban versus rural municipality of residence, province/territory of death, and polysubstance use.

This study only reports substances detected in toxicology or contributing to death based on minimum counts and proportions. In line with privacy and confidentiality guidelines regarding small numbers and potentially identifiable information, all published counts were randomly rounded to base 3, and numbers less than ten were suppressed [19].

## Results

### Specific substances involved in AATDs

There were a total of 7,902 AATDs in Canada from January 1, 2016 to December 31, 2017. Full toxicology reports were available in the majority of coroner and medical examiner files (91%), and among those without a complete toxicology report, 35% had a rapid toxicology report available. Fentanyl was the substance most commonly detected and contributing to AATDs in Canada between 2016 and 2017 and among those cases where fentanyl of known origin contributed to death (51%), 94% of deaths involved non-pharmaceutical fentanyl (Table 1). Fentanyl contributed to death in 97% of cases when detected and contributed to death alone in 19% of AATDs. Cocaine (35%), ethanol (22%), methamphetamine (22%) and morphine (14%) were among the top 5 substances contributing to AATDs. THC (tetrahydrocannabinol) was detected in more than 5% of cases but contributed to death in fewer than 1% of cases (detected *n* = 467; contributing to death *n* = 39). Acetaminophen contributed to 1% of all AATDs but was the sole contributor in 55% of cases where it contributed to death (detected *n* = 679; contributing to death *n* = 87). Carfentanil contributed to 6% of all AATDs during the two-year period and contributed to death in 99% of the cases where detected (detected *n* = 439; contributing to death *n* = 435). The following substances were detected in more than 5% of AATDs but did not contribute to more than 5% of AATDs: codeine, diphenhydramine, diazepam, clonazepam, gabapentin, quetiapine, acetaminophen, citalopram and THC. Among pharmaceutical substances that contributed to or were detected in at least 5% of AATDs, methadone (7%) and hydromorphone (6%) were most often found to be accessed through a diverted prescription and quetiapine was the most common prescribed substance. Amphetamine, which may be a possible metabolite of methamphetamine, most often had an unknown origin.

**Table 1:** Substances involved in 5% or more accidental acute toxicity deaths in Canada, 2016–2017

Substance	Detected (*N* = 7902)	CTD (*N* = 7902)	CTD when detected	CTD alone	Non-pharmaceutical origin when substance CTD	% Pharmaceutical origin when substance CTD				% Unknown origin when substance CTD
						**Total**	**Prescribed**	**Diverted**	**Unknown source**	
**Fentanyl**	3801 (48%)	3717 (47%)	3690 (97%)	699 (19%)	1770 (48%)	120 (3%)	90 (2%)	13 (≤ 1%)	16 (≤ 1%)	1827 (49%)
**Cocaine**	3285 (42%)	2769 (35%)	2745 (84%)	438 (16%)	2769 (100%)	NA	NA	NA	NA	NA
**Ethanol (alcohol)**^**a**^	2641 (33%)	1719 (22%)	1692 (64%)	237 (14%)	NA	NA	NA	NA	NA	NA
**Methamphetamine**	2044 (26%)	1704 (22%)	1692 (83%)	165 (10%)	1704 (100%)	NA	NA	NA	NA	NA
**Morphine**^**b**^	1775 (22%)	1119 (14%)	1110 (63%)	63 (6%)	184 (16%)	124 (11%)	98 (9%)	12 (≤ 1%)	14 (≤ 1%)	811 (73%)
**Amphetamine**^**b**^	1770 (22%)	975 (12%)	972 (55%)	Sup	184 (19%)	15 (2%)	15 (2%)	0 (0%)	0 (0%)	778 (80%)
**Diacetylmorphine**^**c**^	937 (12%)	822 (10%)	795 (85%)	30 (4%)	822 (100%)	NA	NA	NA	NA	NA
**Methadone**	811 (10%)	648 (8%)	645 (80%)	144 (22%)	NA	648 (100%)	222 (34%)	47 (7%)	381 (59%)	NA
**Hydromorphone**	797 (10%)	489 (6%)	486 (61%)	84 (17%)	NA	489 (100%)	180 (37%)	28 (6%)	285 (58%)	NA
**Oxycodone**	675 (9%)	471 (6%)	468 (69%)	60 (13%)	NA	472 (100%)	186 (39%)	18 (4%)	266 (57%)	NA
**Carfentanil**	439 (6%)	435 (6%)	435 (99%)	99 (23%)	435 (100%)	NA	NA	NA	NA	NA
**Codeine**	682 (9%)	321 (4%)	321 (47%)	18 (6%)	18 (6%)	199 (62%)	114 (36%)	Sup	80 (25%)	104 (32%)
**Diphenhydramine**	586 (7%)	195 (2%)	195 (33%)	Sup	NA	195 (100%)	NA	NA	NA	NA
**Diazepam**	585 (7%)	192 (2%)	192 (33%)	0 (0%)	NA	192 (100%)	53 (28%)	Sup	135 (70%)	NA
**Clonazepam**	802 (10%)	144 (2%)	141 (18%)	0 (0%)	NA	144 (100%)	66 (46%)	Sup	72 (50%)	NA
**Gabapentin**	413 (5%)	123 (2%)	123 (30%)	0 (0%)	NA	123 (100%)	51 (42%)	Sup	65 (53%)	NA
**Quetiapine**	490 (6%)	96 (1%)	96 (20%)	Sup	NA	96 (100%)	57 (60%)	Sup	36 (38%)	NA
**Acetaminophen**	679 (9%)	87 (1%)	75 (11%)	48 (55%)	NA	87 (100%)	NA	NA	NA	NA
**Citalopram or escitalopram**	471 (6%)	75 (1%)	78 (17%)	Sup	NA	75 (100%)	30 (38%)	Sup	46 (61%)	NA
**THC**	467 (6%)	39 (≤ 1%)	39 (8%)	Sup	10 (24%)	0 (0%)	0 (0%)	0 (0%)	0 (0%)	31 (76%)

*Abbreviations*: *CTD* Contributed to death, *NA* Not applicable, *THC* Tetrahydrocannabinol, *Sup* Suppressed

Table is arranged in descending order based on CTD column (column two). Source and origin cells listed as not applicable indicate substances that were only known to be pharmaceutical, non-pharmaceutical or over the counter pharmaceuticals, and therefore were not distinguished as prescribed or diverted during the study period. More than one substance may be involved in an accidental acute toxicity death, and a substance could be acquired from more than one origin or source, therefore proportions are not mutually exclusive. Data from British Columbia were only available for people who experienced accidental acute toxicity deaths involving unregulated drugs and/or drugs sold illicitly. Thus, data for people who experienced acute toxicity deaths due solely to prescribed substances or alcohol were not available. Data on the source of substances involved was not available for British Columbia. Data on the source and origin of substances that could be of either pharmaceutical or non-pharmaceutical origin (e.g., fentanyl, amphetamine, morphine, codeine, and THC) was not available for all British Columbia deaths. All substances listed for British Columbia were substances detected that were deemed relevant to death and therefore were listed as both detected and contributing to death which may result in a bias in the %CTD when detected (column 3) value to be closer to 100%

^a^Alcohol may be detected due to consumption or post-mortem endogenous ethanol production and its detection should be interpreted with caution

^b^Amphetamine is a methamphetamine metabolite, and morphine is a diacetylmorphine (heroin) metabolite. Their presence in toxicology tests suggests consumption of either the metabolites or their parent substances

^c^Diacetylmorphine is commonly known as heroin. All values (except the %CTD when detected column values) represent minimum proportions. Cells with counts less than 10 are suppressed to protect privacy. Counts are randomly rounded to base three. Percentages are based on rounded counts

Community of residence type was unknown for 5.6% of all AATDs. Fentanyl was the most commonly detected substance and most often contributed to AATDs across all residence community types (urban [detected 49%; contributing to death 48%], rural [detected 34%; contributing to death 32%], and AATDs where community of residence type was unknown [detected and contributed to death 62%]) (Table 2). Methamphetamine was the second most common substance contributing to death among people who died while residing in areas where the community type of residence was unknown (42%); cocaine was the second most common substance detected (43%) and contributing to death (36%) among people who died while residing in urban communities (43%) and ethanol was the second most common substance detected (40%) and contributing to death (28%) among people who died while residing in rural communities (33%). Ethanol and hydromorphone contributed to a higher proportion of AATDs among people who died while residing in rural communities than those in urban communities or those whose community type was unknown.

**Table 2:** Substances involved in 10% or more accidental acute toxicity deaths by community of residence type

Community of residence type	Urban (*N* = 6591)	Rural (*N* = 885)	Unknown (*N* = 426)
**Detected**			
Fentanyl	3240 (49%)	297 (34%)	264 (62%)
Methamphetamine	1671 (25%)	177 (20%)	195 (46%)
Amphetamine^a^	1446 (22%)	144 (16%)	180 (42%)
Cocaine	2817 (43%)	294 (33%)	174 (41%)
Ethanol (alcohol)^b^	2151 (33%)	357 (40%)	135 (32%)
Morphine^a^	1485 (23%)	174 (20%)	114 (27%)
Diacetylmorphine (heroin)	801 (12%)	60 (7%)	75 (18%)
Methadone	705 (11%)	72 (8%)	36 (8%)
Codeine	549 (8%)	108 (12%)	24 (6%)
Clonazepam	696 (11%)	81 (9%)	21 (5%)
Hydromorphone	639 (10%)	138 (16%)	18 (4%)
Oxycodone	564 (9%)	96 (11%)	18 (4%)
Acetaminophen	540 (8%)	126 (14%)	15 (4%)
Diphenhydramine	483 (7%)	90 (10%)	Sup
**Contributing to death**			
Fentanyl	3171 (48%)	282 (32%)	264 (62%)
Cocaine	2373 (36%)	243 (27%)	153 (36%)
Methamphetamine	1389 (21%)	135 (15%)	180 (42%)
Ethanol (alcohol)^b^	1368 (21%)	246 (28%)	108 (25%)
Morphine	921 (14%)	105 (12%)	93 (22%)
Amphetamine^a^	768 (12%)	63 (7%)	144 (34%)
Diacetylmorphine (heroin)	705 (11%)	51 (6%)	63 (15%)
Hydromorphone	387 (6%)	93 (11%)	12 (3%)

*Abbreviations*: *Sup* Suppressed

Urban municipalities include census metropolitan areas (CMAs) with populations of at least 100,000 residents and census agglomerations (CAs) with populations of at least 10,000 residents. Rural municipalities are defined as all areas outside of CMAs and CAs. 5.6% of people from the study who died of accidental acute toxicity could not be linked to the area-based variables that measured community size and remoteness due to missing location of residence information at the time of their death and have been grouped under the unknown community of residence type category. A disproportionate number of those who could not be linked were unhoused at the time of their death (60%) while others in this category may include people who were travelling outside their home province, postal codes that did not match with residential areas as designated by Statistics Canada, missing data or data entry errors. Data from British Columbia were only available for people who experienced accidental acute toxicity deaths involving unregulated drugs and/or drugs sold illicitly. As such, data for people who experienced acute toxicity deaths due solely to prescribed substances or alcohol were not available. All substances listed for British Columbia were substances detected that were deemed relevant to death and therefore were listed as both detected and contributing to death

^a^Amphetamine is a methamphetamine metabolite, and morphine is a diacetylmorphine (heroin) metabolite. Their presence in toxicology tests suggests consumption of either the metabolites or their parent substances

^b^Alcohol may be detected due to consumption or post-mortem endogenous ethanol production and its detection should be interpreted with caution. All values reported represent minimum proportions. Cells with counts less than 10 are suppressed to protect privacy. Counts are randomly rounded to base three. Percentages are based on rounded counts

Neighbourhood income quintile was unknown for 27% of all AATDs. Fentanyl and cocaine were the top two substances detected and contributing to death for all neighbourhood income quintiles (Table 3). Compared to other quintiles, fentanyl, methamphetamine, morphine, amphetamine, and diacetylmorphine (heroin) contributed to the highest proportion of AATDs for those whose neighbourhood income quintile was unknown. Methadone contributed to a higher proportion of AATDs among those living in the lowest (11%) and medium–low (10%) neighbourhood income quintiles compared to others and ethanol contributed to a higher proportion of AATDs in the highest (24%) and medium–high (24%) neighbourhood income quintiles. Hydromorphone, acetaminophen, codeine, clonazepam and oxycodone were often detected but did not contribute to 10% or more of AATDs among any of the income quintile groups.

**Table 3:** Substances involved in 10% or more accidental acute toxicity deaths by neighborhood income quintile

Neighbourhood income quintile after-tax	Q1 (lowest) (*N* = 2499)	Q2 (medium–low) (*N* = 1251)	Q3 (middle) (*N* = 915)	Q4 (medium–high) (*N* = 744)	Q5 (highest) (*N* = 558)	Unknown (*N* = 1929)
**Detected**						
Fentanyl	1074 (43%)	567 (45%)	429 (47%)	366 (49%)	252 (45%)	1110 (58%)
Cocaine	1005 (40%)	489 (39%)	393 (43%)	336 (45%)	243 (44%)	819 (42%)
Ethanol (alcohol)^a^	864 (35%)	450 (36%)	303 (33%)	279 (38%)	213 (38%)	534 (28%)
Methamphetamine	687 (27%)	276 (22%)	189 (21%)	108 (15%)	84 (15%)	702 (36%)
Morphine^b^	576 (23%)	264 (21%)	186 (20%)	144 (19%)	114 (20%)	492 (26%)
Amphetamine^b^	564 (23%)	240 (19%)	162 (18%)	105 (14%)	87 (16%)	612 (32%)
Methadone	333 (13%)	153 (12%)	78 (9%)	60 (8%)	39 (7%)	147 (8%)
Clonazepam	318 (13%)	135 (11%)	96 (10%)	81 (11%)	54 (10%)	120 (6%)
Hydromorphone	315 (13%)	132 (11%)	102 (11%)	69 (9%)	60 (11%)	117 (6%)
Codeine	282 (11%)	105 (8%)	75 (8%)	57 (8%)	45 (8%)	117 (6%)
Acetaminophen	282 (11%)	123 (10%)	81 (9%)	72 (10%)	36 (6%)	84 (4%)
Diacetylmorphine (heroin)	252 (10%)	126 (10%)	96 (10%)	69 (9%)	60 (11%)	330 (17%)
Oxycodone	222 (9%)	129 (10%)	105 (11%)	72 (10%)	51 (9%)	93 (5%)
**Contributed to death**						
Fentanyl	1044 (42%)	555 (44%)	417 (46%)	354 (48%)	246 (44%)	1101 (57%)
Cocaine	828 (33%)	402 (32%)	321 (35%)	282 (38%)	201 (36%)	735 (38%)
Methamphetamine	558 (22%)	228 (18%)	147 (16%)	87 (12%)	72 (13%)	612 (32%)
Ethanol (alcohol)^a^	513 (21%)	261 (21%)	201 (22%)	177 (24%)	135 (24%)	432 (22%)
Morphine^b^	330 (13%)	150 (12%)	111 (12%)	84 (11%)	72 (13%)	369 (19%)
Methadone	273 (11%)	129 (10%)	54 (6%)	45 (6%)	33 (6%)	120 (6%)
Amphetamine^b^	264 (11%)	105 (8%)	78 (9%)	45 (6%)	48 (9%)	435 (23%)
Diacetylmorphine (heroin)	216 (9%)	105 (8%)	81 (9%)	57 (8%)	54 (10%)	309 (16%)

Neighbourhood income quintiles are calculated based on income after-tax at the Dissemination Area (DA) level. Quintile 1 is the lowest neighbourhood income quintile and quintile 5 is the highest. 27% of people from the study who died of accidental acute toxicity could not be linked with PCCF + data to identify their neighbourhood-income level based on missing postal code of residence information at the time of their death and have been grouped in the unknown income quintile category. A disproportionate number of those who could not be linked were unhoused at the time of their death (25%) while others in this category may include people who were travelling outside their home province, postal codes that did not match with residential areas as designated by Statistics Canada, missing data or data entry errors. Data from British Columbia were only available for people who experienced accidental acute toxicity deaths involving unregulated drugs and/or drugs sold illicitly. As such, data for people who experienced acute toxicity deaths due solely to prescribed substances or alcohol were not available. All substances listed for British Columbia were substances detected that were deemed relevant to death and therefore were listed as both detected and contributing to death

^a^Alcohol may be detected due to consumption or post-mortem endogenous ethanol production and its detection should be interpreted with caution

^b^Amphetamine is a methamphetamine metabolite, and morphine is a diacetylmorphine (heroin) metabolite. Their presence in toxicology tests suggests consumption of either the metabolites or their parent substances. Cells with counts less than 10 are suppressed to protect privacy. Counts are randomly rounded to base three. Percentages are based on rounded counts

### Substances that emerged or declined over 2016 to 2017

**Fig. 1:**
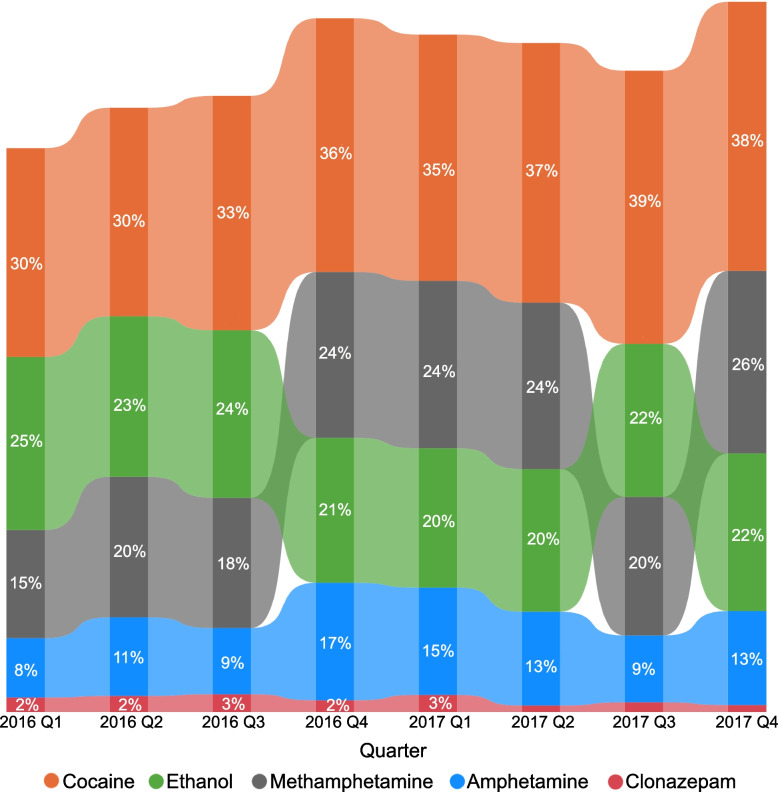
Quarterly proportion of select non-opioid substances contributing to accidental acute toxicity deaths, Canada, 2016–2017. Note: Substances presented in this chart are non-opioids that contributed to a proportion of AATDs per quarter outside the control limits of the baseline range of the first three quarters of 2016 based on 3 standard deviations and contributed to more than 10 deaths overall. Xylazine, pseudoephedrine, gamma-hydroxybutyrate and duloxetine have been excluded from this chart due to suppression of cell counts less than 10 to protect privacy. Proportions are based on counts randomly rounded to base three. Size of ribbons indicate proportions and location of ribbons indicate rank (bars on the top are ranked highest and bars at the bottom are ranked lowest based on proportions of deaths the substance contributed to per quarter). All values reported represent minimum proportions. Amphetamine is a methamphetamine metabolite. Its presence in toxicology testing could indicate that either it or its parent substance had been consumed

The first three quarters of 2016 represent the time period immediately before the public health crisis was declared in Canada. During these three quarters, cocaine contributed to 30–33% of AATDs per quarter, and methamphetamine contributed to 15–18% of AATDs per quarter (Fig. 1). The proportion of AATDs per quarter where methamphetamine, cocaine and amphetamine were the contributors increased during the fourth quarter of 2016, and this increase persisted until the end of the study period for cocaine but temporarily dipped back to the starting range for methamphetamine and amphetamine during the third quarter of 2017. The proportion of AATDs per quarter with clonazepam as contributor decreased during the second and fourth quarters of 2017. Several substances that contributed to a small proportion of deaths (≤ 1% per quarter and were excluded from the ribbon chart to protect privacy) had temporary increases in the proportion of AATDs they contributed to during this period, including duloxetine during the second and third quarters of 2016; xylazine during the fourth quarter of 2016; pseudoephedrine during the last quarter of 2016 and first quarter of 2017, and gamma hydroxybutyrate (GHB) in the second quarter of 2017.

**Fig. 2:**
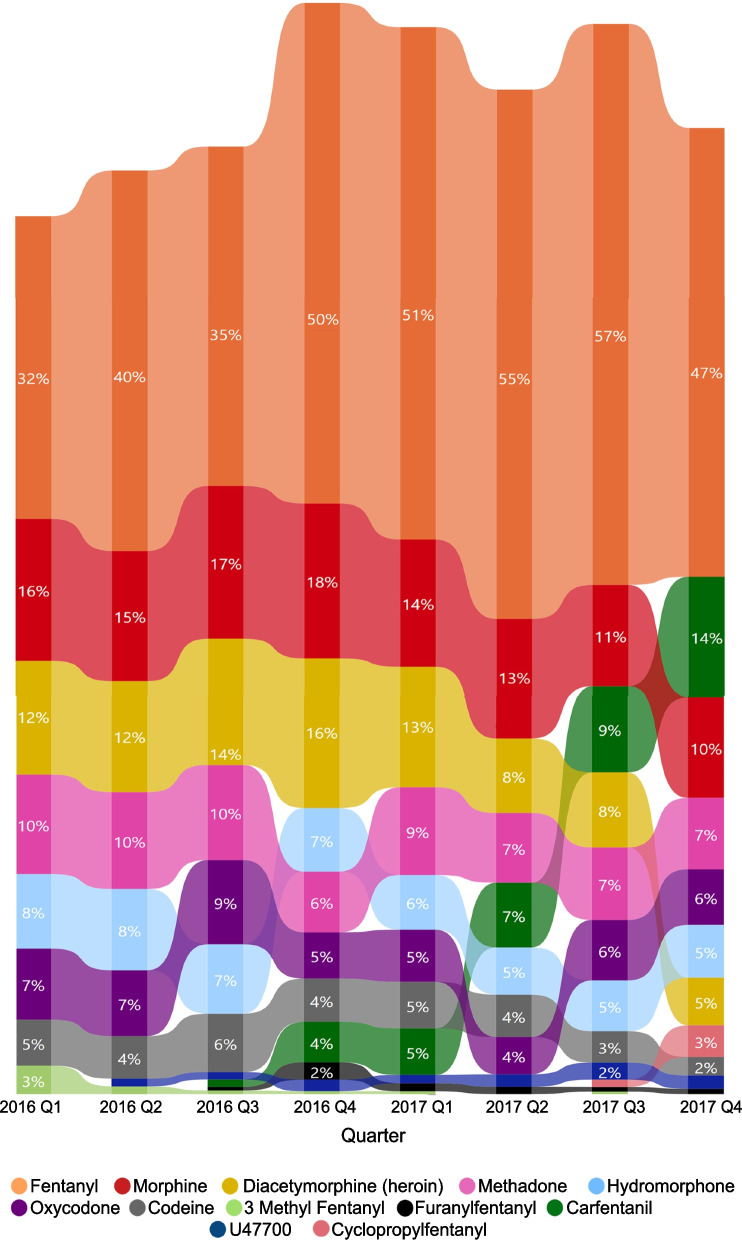
Quarterly proportion of select opioid substances contributing to accidental acute toxicity deaths, Canada, 2016–2017. Note: Substances presented in this chart are opioids that contributed to a proportion of AATDs per quarter outside the control limits of the baseline range of the first three quarters of 2016 based on 3 standard deviations and contributed to more than 10 deaths overall. Buprenorphine has been excluded and ribbons with proportions not reported have been suppressed due to cell counts of less than 10 to protect privacy. Proportions are based on counts randomly rounded to base three. Size of ribbons indicate proportions and location of ribbons indicate rank (bars on the top are ranked highest and bars at the bottom are ranked lowest based on proportions of deaths the substance contributed to per quarter). All values reported represent minimum proportions

Among opioids, diacetylmorphine (heroin) saw the steepest decrease and carfentanil saw the steepest increase in the proportion of AATDs these substances contributed to per quarter between 2016 and 2017 (Fig. 2). The proportion of AATDs per quarter with carfentanil as a contributor quickly rose over the study period starting in the second quarter of 2016 and continued to increase each quarter until the end of the study period. While fentanyl remained the number one substance contributing to AATDs throughout the study period, there was a notable increase in the proportion of AATDs it contributed to in the fourth quarter of 2016, which persisted over the two year period. Proportions of AATDs per quarter with morphine, hydromorphone, methadone and codeine contributing to death decreased towards the end of the study period. 3-Methylfentanyl contributed to a deceasing number of deaths over the first three quarters of 2016 and did not contribute to death in subsequent quarters. Cyclopropyl/crotonyl fentanyl emerged as a new substance contributing to AATDs during the third quarter of 2017. Buprenorphine emerged as a new substance contributing to AATDs (not shown as it contributed to ≤ 1% AATDs per quarter) during the first quarter of 2017.

### Involvement of multiple substances

For most AATDs, multiple substances were detected (89%) and contributed to death (70%) (Table 4). These proportions were similar among males and females. While the proportion of AATDs with multiple substances detected stayed the same in 2016 and 2017 (89%), we observed a 3% increase in the proportion of cases with multiple substances contributing to the death, from 68% in 2016 to 71% in 2017. Multiple substances contributed to death most often in British Columbia (83%) and least often in the Prairie region (56%). Multiple substances contributed to the highest proportions of AATDs among those aged 20 to 49 years (ranging from 70 to 72%) and the lowest proportion of AATDs among those aged 70 + (52%). While multiple substances were detected in similar proportions among those in urban, rural and unknown community types, the latter had the highest proportions of AATDs with multiple substances contributing to death (79%). Multiple substances contributed to death in the highest proportion among those living in neighbourhoods where the neighbourhood income quintile was unknown (76%) and in lowest proportions among those living in neighbourhoods with a medium–high (Q4) income quintile (65%). However, multiple substances were detected in the lowest proportion among those whose income quintile was unknown (86%) and the highest proportion among those living in the lowest (Q1), medium to low (Q2) and medium (Q3) income quintile neighbourhoods (90%).

**Table 4:** Proportions of accidental acute toxicity deaths involving multiple substances by socioeconomic, sociodemographic and geographic factors

Characteristic	Number	Multiple substances detected	Single substance detected	Unknown number of substances detected	Multiple substances CTD	Single substance CTD	Unknown number of substances CTD
**Total**	7902	7035 (89%)	675 (9%)	189 (2%)	5514 (70%)	2262 (29%)	126 (2%)
**Sex**							
Female	2016	1821 (90%)	147 (7%)	48 (2%)	1401 (69%)	588 (29%)	27 (≤ 1%)
Male	5886	5214 (89%)	528 (9%)	141 (2%)	4113 (70%)	1674 (28%)	99 (2%)
**Province or region**							
British Columbia	2469	2031 (82%)	309 (13%)	129 (5%)	2055 (83%)	318 (13%)	96 (4%)
Alberta	1506	1425 (95%)	69 (5%)	15 (≤ 1%)	1050 (70%)	450 (30%)	Sup
Prairie region^a^	465	432 (93%)	30 (6%)	6 (≤ 1%)	261 (56%)	201 (43%)	Sup
Ontario	2,475	2235 (90%)	225 (9%)	18 (≤ 1%)	1533 (62%)	939 (38%)	Sup
Quebec	678	633 (94%)	30 (4%)	15 (2%)	408 (60%)	264 (39%)	Sup
Atlantic region^a^	282	261 (93%)	12 (4%)	Sup	189 (67%)	78 (28%)	15 (5%)
Northern region^a^	27	24 (89%)	Sup	0 (0%)	18 (67%)	Sup	0 (0%)
**Year of death**							
2016	3372	3003 (89%)	288 (9%)	81 (2%)	2301 (68%)	1026 (30%)	42 (≤ 1%)
2017	4530	4035 (89%)	384 (8%)	108 (2%)	3213 (71%)	1236 (27%)	81 (2%)
**Age group**							
≥ 19 years	171	135 (78%)	30 (17%)	Sup	105 (61%)	60 (35%)	Sup
20–29 years	1461	1308 (90%)	129 (9%)	27 (2%)	1029 (70%)	414 (28%)	18 (≤ 1%)
30–39 years	2103	1908 (91%)	150 (7%)	45 (2%)	1509 (72%)	564 (27%)	33 (2%)
40–49 years	1722	1557 (90%)	117 (7%)	48 (3%)	1248 (72%)	438 (25%)	36 (2%)
50–59 years	1740	1539 (88%)	165 (9%)	36 (2%)	1191 (68%)	522 (30%)	24 (≤ 1%)
60–69 years	609	525 (86%)	69 (11%)	15 (2%)	381 (63%)	216 (36%)	Sup
≥ 70 years	99	72 (73%)	15 (15%)	12 (12%)	51 (52%)	48 (48%)	0 (0%)
**Community type of residence**							
Urban	6591	5871 (89%)	570 (9%)	150 (2%)	4587 (70%)	1908 (29%)	96 (≤ 1%)
Rural	885	789 (89%)	84 (9%)	15 (2%)	591 (67%)	285 (32%)	12 (≤ 1%)
Unknown	426	378 (89%)	21 (5%)	24 (6%)	336 (79%)	72 (17%)	18 (4%)
**Area-based neighbourhood income quintile after-tax**							
Q1 (lowest)	2499	2262 (90%)	201 (8%)	36 (≤ 1%)	1746 (70%)	726 (29%)	24 (≤ 1%)
Q2 (medium to low)	1,251	1131 (90%)	99 (8%)	21 (2%)	831 (66%)	405 (32%)	15 (≤ 1%)
Q3 (medium)	915	819 (90%)	78 (9%)	18 (2%)	609 (67%)	294 (32%)	12 (≤ 1%)
Q4 (medium to high)	744	657 (88%)	75 (10%)	12 (2%)	486 (65%)	249 (33%)	12 (2%)
Q5 (highest)	558	495 (89%)	54 (10%)	12 (2%)	369 (66%)	186 (33%)	Sup
Unknown	1929	1668 (86%)	171 (9%)	90 (5%)	1473 (76%)	399 (21%)	57 (3%)

*Abbreviations*: *CTD* Contributed to death, *Sup* Suppressed

^a^The Prairie region represents Saskatchewan and Manitoba. The Atlantic region represents Nova Scotia, Newfoundland and Labrador, Prince Edward Island and New Brunswick. The Northern region represents Nunavut, Yukon Territories and the Northwest Territories. Counts less than 10 are suppressed to protect privacy. Counts are randomly rounded to base three. Percentages are based on rounded counts and all reported values represent minimum proportions. Data from British Columbia were only available for people who experienced accidental acute toxicity deaths involving unregulated drugs and/or drugs sold illicitly. Therefore, data for people who experienced acute toxicity deaths due solely to prescribed substances or alcohol were unavailable. 5.6% of people from the study who died of accidental acute toxicity could not be linked to the area-based variables due to missing location of residence information and have been grouped under the unknown community of residence type category. Quintile 1 is the lowest neighbourhood income quintile and quintile 5 is the highest. 27% of people from the study who died of accidental acute toxicity could not be linked with PCCF + data to identify their neighbourhood-income level based on missing postal code of residence information at the time of their death and have been grouped in the unknown income quintile category. A disproportionate number of those who could not be linked were unhoused at the time of their death (unknown community type 60%; unknown neighbourhood income quintile 25%) while others in this category may include people who were travelling outside their home province, postal codes that did not match with residential areas as designated by Statistics Canada, missing data or data entry errors

### Top substances, substance combinations and substance classes contributing to death

**Fig. 3:**
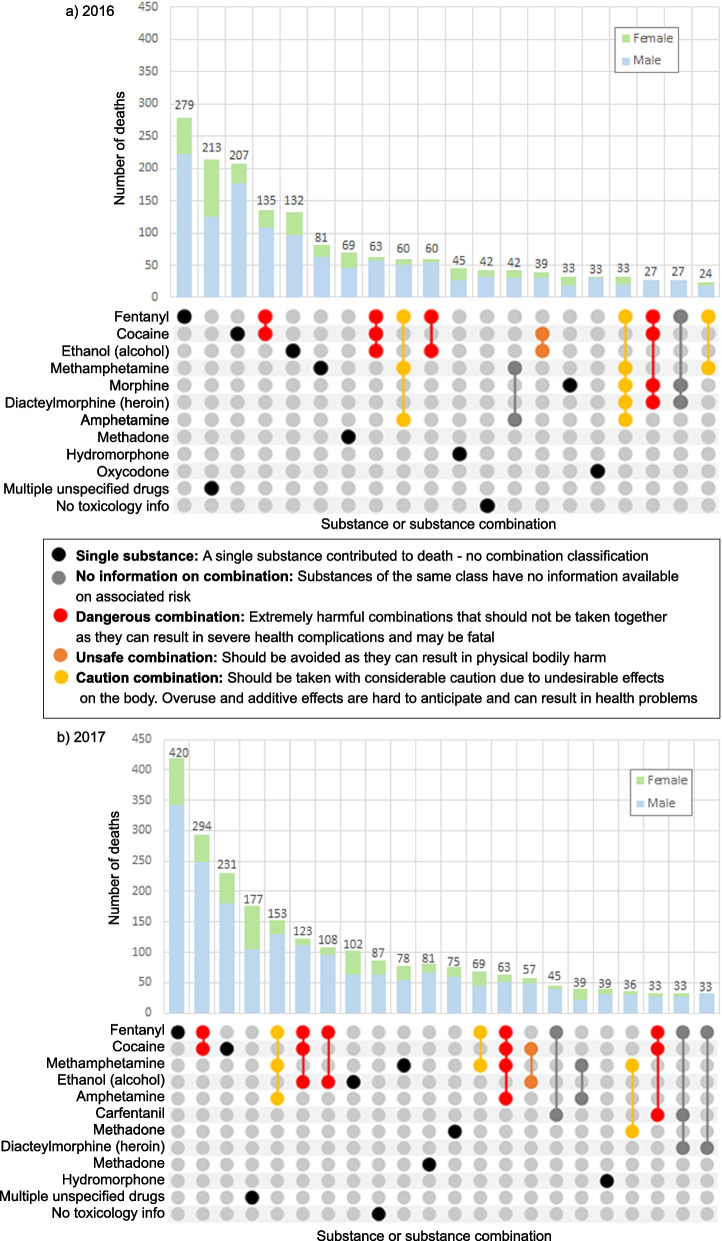
Top 20 substances/substance combinations contributing to accidental acute toxicity deaths in Canada, (**a**) 2016, (**b**) 2017. Notes: All panels have two segments each: (1) The grids display specific combinations of substances causing death in vertical columns. Substances causing death are represented by a coloured dot joined to other substances in the combination by a line. Grid combinations have been coloured to indicate their safety levels based on Tripsit Wiki’s Guide to Drug combinations, while single substances remained black [29]. Grid colour coding indicates safety only based on the types of substances involved, not the number. As more substances are involved, the actual risk level may increase, regardless of whether substance combinations are safe or not due to additive effects of the substances ingested, thus results should be interpreted with caution. (2) The vertical bar chart shows the number of deaths caused by a specific substance/substance combination described in the grid below. These bars are coloured to show the number of deaths involving each sex, and the number on top of each bar represents the total number of deaths involving the substance/substance combination identified in the grid. Multiple unspecified drugs refers to cases where multiple substances contributed to death but the specific substances were not identified. ‘No toxicology information available’ refers to cases which have no information on specific substances involved or the number of substances involved in death. Amphetamine is a metabolite of methamphetamine and morphine is a metabolite of diacetylmorphine (heroin). Their presence in toxicology testing could indicate that either they or their parent substance had been consumed. Counts are randomly rounded to base 3 and numbers reported are minimum counts

Fentanyl alone, cocaine alone, the combination of these two substances and multiple unspecified drugs were the leading causes of AATDs in both 2016 and 2017 (Fig. 3). All combinations classified as dangerous according to the TripSit guide in the UpSet plots for both 2016 and 2017 involved fentanyl (an opioid). Stimulants, alcohol or both often contributed to death with fentanyl in these combinations. Combinations of opioids with methamphetamine and/or amphetamine were three of the top 20 combinations contributing to death in both 2016 and 2017 despite only being classified at a “cautionary” risk level by the TripSit guide. Among the top 20 combinations, the combination of cocaine and ethanol (alcohol) was the only combination classified as “unsafe” by the TripSit guide across both years and contributed to a total of 96 AATDs during this time. All substances and combinations contributed to a higher number of male AATDs than female AATDs in both years except morphine, which contributed to a similar number of male (55%) and female (45%) AATDs in 2016.

**Fig. 4:**
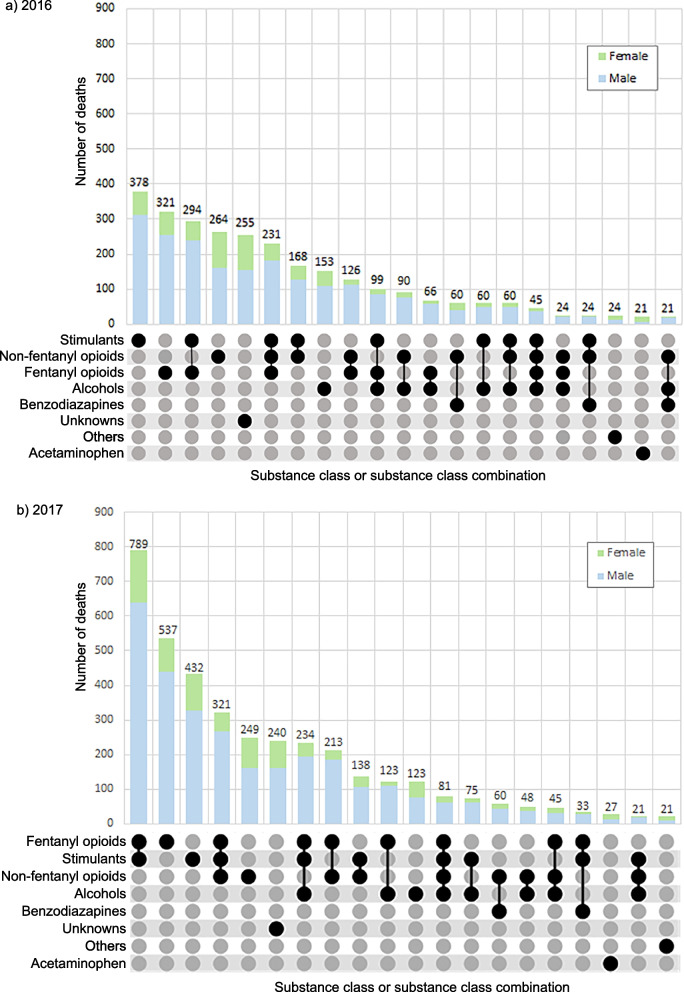
Top 20 substance classes/substance class combinations contributing to accidental acute toxicity deaths in Canada, (**a**) 2016, (**b**) 2017**.** Notes: Each panel has two segments: (1) The grids display specific combinations of substance classes contributing to death in vertical columns. Substance classes contributing to death are represented by a black dot joined to other substance classes in the combination by a line. (2) The vertical bar chart shows the number of deaths where a specific substance class/substance class combination described in the grid below contributed. These bars are coloured to show the number of deaths involving each sex, and the number on top of each bar represents the total number of deaths involving the substance class/substance class combination identified in the grid. Unknown refers to cases where the cause of death was listed as substance related with no specific substances specified. A complete list of substances in each class can be found in Additional file 1

Stimulants, fentanyl opioids, non-fentanyl opioids, alcohol and the various combinations involving these classes were the leading contributors of AATDs in both 2016 and 2017 (Fig. 4). Benzodiazepines were among the top 20 leading contributors of AATDs, but only in combination with non-fentanyl opioids or non-fentanyl opioids with stimulants and/or alcohol. Acetaminophen alone as well as the others class alone are the only substance classes among the top 20 contributors to death that impacted females more than males.

There are notable differences in the substances/substance combinations contributing to more than 10 AATDs at the provincial and regional levels [see Additional file 4]. British Columbia had the highest frequency and greatest diversity of substance combinations contributing to more than 10 AATDs. It also had as many as 6 substances in combination contributing to AATDs, which is the highest among all provinces. Fentanyl and cocaine were the only two substances that contributed to death alone in more than 10 AATDs in British Columbia. Ontario and Alberta had many combinations contributing to more than 10 AATDs, but fewer than British Columbia. Several fentanyl-opioids and non-fentanyl opioids as well as acetaminophen were among the top substances that contributed to more than 10 AATDs alone in Alberta and Ontario. For Quebec, the Prairie region and the Atlantic region, the only substance combination contributing to 10 or more AATDs was 'multiple unspecified drugs'. Ethanol, cocaine, carfentanil, methadone, fentanyl and methamphetamine were the top substances contributing to deaths alone in the Prairie region, cocaine, methamphetamine ethanol and fentanyl were the top substances contributing to deaths alone in Quebec and cocaine and ethanol were the top substances contributing to deaths alone in the Atlantic region. The Atlantic region had 10 or more AATDs with no information on specific substances or the number of substances contributing to death. In the Northern region, no substance or substance combination resulted in greater than 10 deaths.

### Source and origin of substances contributing to death

**Fig. 5:**
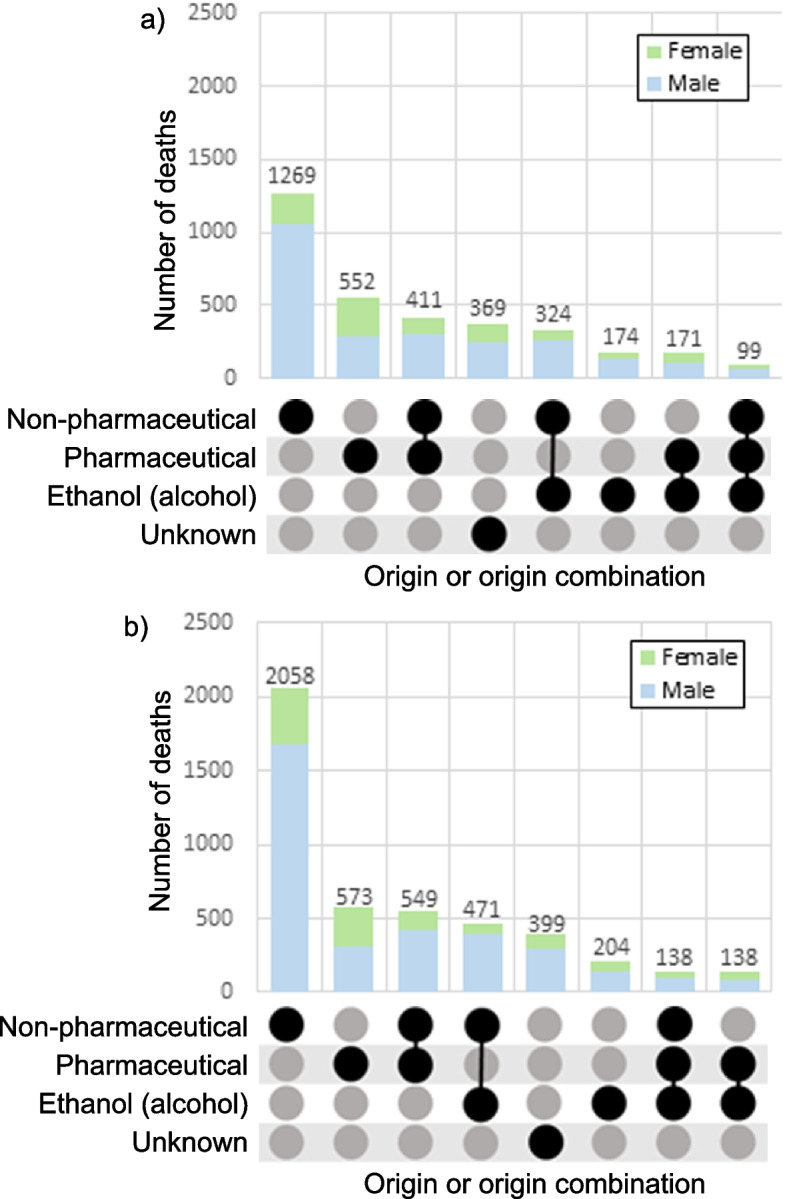
Accidental acute toxicity deaths by origin of substances contributing to deaths in Canada, (**a**) 2016, (**b**) 2017. Notes: Only deaths due to illicit substances (unregulated drugs and/or drugs sold illicitly) were available from British Columbia, and therefore pharmaceutical only and alcohol only deaths will be undercounted. Data on the source and origin of substances that could be of either pharmaceutical or non-pharmaceutical origin (e.g. fentanyl, amphetamine, morphine, codeine, and THC) were not available for all British Columbia cases

For both study years, people who died from accidental acute toxicity most often had exclusively non-pharmaceutical substances contributing to their deaths and the majority of these deaths involved males (Fig. 5). Exclusively pharmaceutical substances were the next most common origin of substances contributing to death among people who died from accidental acute toxicity. Females died of accidental acute toxicities involving exclusively pharmaceutical substances more commonly than any other origin pattern in 2016, but that changed to exclusively non-pharmaceutical substances in 2017. There were more AATDs in 2017 where the combination of ethanol, non-pharmaceutical, and pharmaceutical substances contributed to the death than in 2016.

The 30 to 39 years old age group represented the biggest proportion of people who died of accidental acute toxicity when a non-pharmaceutical substance contributed to death (29%) and when a diverted pharmaceutical contributed to death (29%) (Table 5). In contrast, the 50 to 59 years old age group represented the biggest proportion of people who died of accidental acute toxicity when a pharmaceutical substance contributed to death (27%). Regional trends showed that AATDs where non-pharmaceutical substances contributed to death were most common in British Columbia (36%), AATDs where pharmaceutical substances contributed to death were most common in Ontario (40%) and AATDs where diverted pharmaceutical substances contributed to death were most common in Prairie region (29%) and Ontario (29%). While most females and males who had diverted pharmaceuticals contributing to their deaths resided in the lowest (Q1) income quintile neighbourhoods, female deaths were more concentrated in the lower income quintiles than males. Female deaths where diverted pharmaceuticals contributed to death occurred at a higher proportion in 2017 than 2016.

**Table 5:** Source/origin of substances involved in accidental acute toxicity deaths by socioeconomic, sociodemographic and geographic factors

**Characteristic**	**Non-pharmaceutical CTD**			**Pharmaceutical CTD**			**Pharmaceutical detected or CTD**			**Diverted pharmaceutical CTD**			**Diverted pharmaceutical detected or CTD**		
	**Female**	**Male**	**All**	**Female**	**Male**	**All**	**Female**	**Male**	**All**	**Female**	**Male**	**All**	**Female**	**Male**	**All**
**Total**	1059 (20%)	4266 (80%)	5325(100%)	945 (36%)	1686 (64%)	2628 (100%)	1464 (33%)	3003 (67%)	4470 (100%)	54 (≤ 1%)	96 (≤ 1%)	153 (2%)	75 (≤ 1%)	123 (2%)	198 (3%)
**Age group**															
≤ 19 years	51 (5%)	63 (≤ 1%)	114 (2%)	21 (2%)	30 (2%)	51 (2%)	39 (3%)	54 (2%)	90 (2%)	Sup	Sup	Sup	Sup	Sup	Sup
20 to 29 years	246 (23%)	897 (21%)	1143 (21%)	114 (12%)	282 (17%)	396 (15%)	207 (14%)	531 (18%)	738 (17%)	15 (28%)	24 (25%)	39 (25%)	15 (20%)	27 (22%)	42 (21%)
30 to 39 years	303 (29%)	1269 (30%)	1566 (29%)	174 (18%)	396 (23%)	573 (22%)	303 (21%)	813 (27%)	1113 (25%)	18 (33%)	30 (31%)	45 (29%)	24 (32%)	39 (32%)	63 (32%)
40 to 49 years	228 (22%)	954 (22%)	1182 (22%)	189 (20%)	375 (22%)	561 (21%)	312 (21%)	645 (21%)	957 (21%)	12 (22%)	15 (16%)	27 (18%)	15 (20%)	18 (15%)	36 (18%)
50 to 59 years	186 (18%)	831 (19%)	1017 (19%)	291 (31%)	417 (25%)	708 (27%)	396 (27%)	684 (23%)	1080 (24%)	Sup	18 (19%)	24 (16%)	Sup	27 (22%)	33 (17%)
60 to 69 years	42 (4%)	237 (6%)	279 (5%)	123 (13%)	156 (9%)	279 (11%)	168 (11%)	240 (8%)	408 (9%)	Sup	Sup	12 (8%)	Sup	Sup	15 (8%)
≥ 70 years	Sup	18 (≤ 1%)	24 (≤ 1%)	33 (3%)	30 (2%)	66 (3%)	42 (3%)	39 (≤ 1%)	84 (2%)	Sup	Sup	Sup	Sup	Sup	Sup
**Year of death**															
2016	423 (40%)	1683 (39%)	2106 (40%)	462 (49%)	768 (46%)	1230 (47%)	687 (47%)	1323 (44%)	2010 (45%)	24 (44%)	48 (50%)	72 (47%)	42 (56%)	63 (51%)	102 (52%)
2017	633 (60%)	2586 (61%)	3219 (60%)	483 (51%)	915 (54%)	1398 (53%)	777 (53%)	1680 (56%)	2457 (55%)	30 (56%)	48 (50%)	78 (51%)	33 (44%)	63 (51%)	96 (48%)
**Multiple substances contributing to death**															
Yes	825 (78%)	3249 (76%)	4077 (77%)	705 (75%)	1386 (82%)	2091 (80%)	1020 (70%)	2124 (71%)	3144 (70%)	36 (67%)	72 (75%)	108 (71%)	48 (64%)	93 (76%)	141 (71%)
No	231 (22%)	1017 (24%)	1248 (23%)	237 (25%)	300 (18%)	537 (20%)	438 (30%)	870 (29%)	1311 (29%)	18 (33%)	27 (28%)	45 (29%)	24 (32%)	30 (24%)	54 (27%)
Unknown	0 (0%)	0 (0%)	0 (0%)	0 (0%)	0 (0%)	0 (0%)	Sup	Sup	12 (≤ 1%)	0 (0%)	0 (0%)	0 (0%)	Sup	0 (0%)	Sup
**Residence community type**															
Urban	912 (86%)	3618 (85%)	4530 (85%)	774 (82%)	1410 (84%)	2184 (83%)	1200 (82%)	2532 (84%)	3732 (83%)	42 (78%)	78 (81%)	123 (80%)	57 (76%)	99 (80%)	159 (80%)
Rural	81 (8%)	366 (9%)	447 (8%)	150 (16%)	207 (12%)	357 (14%)	222 (15%)	357 (12%)	579 (13%)	12 (22%)	18 (19%)	30 (20%)	15 (20%)	24 (20%)	39 (20%)
Unknown	66 (6%)	282 (7%)	348 (7%)	21 (2%)	66 (4%)	87 (3%)	42 (3%)	114 (4%)	156 (3%)	0 (0%)	0 (0%)	0 (0%)	0 (0%)	Sup	Sup
**Area-based neighbourhood income quintile after tax**															
Q1 (lowest)	402 (38%)	1215 (28%)	1614 (30%)	378 (40%)	609 (36%)	987 (38%)	579 (40%)	1026 (34%)	1608 (36%)	42 (78%)	42 (44%)	66 (43%)	33 (44%)	51 (41%)	84 (42%)
Q2 (medium–low)	156 (15%)	666 (16%)	819 (15%)	165 (17%)	297 (18%)	462 (18%)	249 (17%)	531 (18%)	780 (17%)	12 (22%)	24 (25%)	36 (24%)	15 (20%)	27 (22%)	45 (23%)
Q3 (middle)	93 (9%)	501 (12%)	594 (11%)	111 (12%)	207 (12%)	318 (12%)	165 (11%)	378 (13%)	540 (12%)	Sup	12 (13%)	15 (10%)	Sup	15 (12%)	24 (12%)
Q4 (medium–high)	99 (9%)	393 (9%)	489 (9%)	102 (11%)	156 (9%)	255 (10%)	153 (10%)	300 (10%)	456 (10%)	Sup	Sup	15 (10%)	Sup	12 (10%)	15 (8%)
Q5 (high)	57 (5%)	321 (8%)	378 (7%)	60 (6%)	123 (7%)	183 (7%)	96 (7%)	243 (8%)	339 (8%)	Sup	Sup	Sup	Sup	Sup	Sup
Unknown	255 (24%)	1176 (28%)	1431 (27%)	126 (13%)	294 (17%)	423 (16%)	222 (15%)	525 (17%)	744 (17%)	Sup	Sup	15 (10%)	Sup	15 (12%)	21 (11%)
**Province or region of death**															
British Columbia	357 (34%)	1551 (36%)	1905 (36%)	135 (14%)	315 (19%)	450 (17%)	135 (9%)	315 (10%)	453 (10%)	0 (0%)	0 (0%)	0 (0%)	0 (0%)	0 (0%)	0 (0%)
Alberta	234 (22%)	918 (22%)	1152 (22%)	177 (19%)	303 (18%)	480 (18%)	312 (21%)	693 (23%)	1005 (22%)	12 (22%)	21 (22%)	33 (22%)	15 (20%)	24 (20%)	39 (20%)
Prairie region^a^	72 (7%)	153 (4%)	228 (4%)	96 (10%)	141 (8%)	234 (9%)	150 (10%)	243 (8%)	393 (9%)	21 (39%)	27 (28%)	45 (29%)	24 (32%)	30 (24%)	54 (27%)
Ontario	342 (32%)	1347 (32%)	1689 (32%)	408 (43%)	717 (43%)	1125 (43%)	588 (40%)	1215 (40%)	1803 (40%)	12 (22%)	33 (34%)	45 (29%)	21 (28%)	36 (29%)	57 (29%)
Quebec	42 (4%)	219 (5%)	261 (5%)	60 (6%)	105 (6%)	165 (6%)	174 (12%)	369 (12%)	540 (12%)	Sup	Supp	12 (8%)	Sup	21 (17%)	27 (14%)
Atlantic region^a^	Sup	69 (2%)	78 (≤ 1%)	63 (7%)	102 (6%)	162 (6%)	96 (7%)	159 (5%)	255 (6%)	Sup	Supp	15 (10%)	Sup	Sup	18 (9%)
Northern region^a^	Sup	12 (≤ 1%)	15 (≤ 1%)	Sup	Sup	Sup	Sup	Sup	15 (≤ 1%)	0 (0%)	Supp	Sup	0 (0%)	Sup	Sup

*Abbreviations*: *CTD* Contributed to death, *Sup* Suppressed

^a^ The Prairie region represents Saskatchewan and Manitoba. The Atlantic region represents Nova Scotia, Newfoundland and Labrador, Prince Edward Island and New Brunswick. The Northern region represents Nunavut, Yukon Territories and the Northwest Territories. Counts less than 10 are suppressed to protect privacy. Counts are randomly rounded to base three. Percentages are based on rounded counts and all reported values represent minimum proportions. Data from British Columbia were only available for people who experienced accidental acute toxicity deaths involving unregulated drugs and/or drugs sold illicitly. Therefore, data for people who experienced acute toxicity deaths due solely to prescribed substances or alcohol were unavailable. 5.6% of people from the study who died of accidental acute toxicity could not be linked to the area-based variables due to missing location of residence information and have been grouped under the unknown community of residence type category. Quintile 1 is the lowest neighbourhood income quintile and quintile 5 is the highest. 27% of people from the study who died of accidental acute toxicity could not be linked with PCCF + data to identify their neighbourhood-income level based on missing postal code of residence information at the time of their death and have been grouped in the unknown income quintile category. A disproportionate number of those who could not be linked were unhoused at the time of their death (unknown community type 60%; unknown neighbourhood income quintile 25%) while others in this category may include people who were travelling outside their home province, postal codes that did not match with residential areas as designated by Statistics Canada, missing data or data entry errors

## Discussion

This is the first Canada-wide study to investigate specific substances and combinations in AATDs and offers key insights into the overdose crisis in Canada. This study examined the substances involved in 7,902 AATDs in Canada that occurred from January 1, 2016 to December 31, 2017. At this time, information on the specific substances detected in toxicology results and their contribution to death is only available through the death investigation files of coroners and medical examiners. In other data sources, the substances involved have been grouped into ICD-10 codes or other analytical categories (e.g., “non-fentanyl opioids”) [5, 7, 8].

While fentanyl opioids, non-fentanyl opioids, and stimulants were the top classes contributing to death; benzodiazepines and other substance classes were also common. This is a significant finding that indicates the importance of expanding overdose death surveillance beyond opioid deaths to encompass all substance-related deaths. In more recent months and years, drug checking data and provincial reports have alerted the public to the contribution of non-pharmaceutical benzodiazepines, such as etizolam, and nitazene opioids to substance-related harms [30–32]. What sets this study apart is its ability to detect newly emerging substances and discover harmful combinations due to its precision and granularity as it is based on toxicology data which does not rely on pre-conceived substance class categories and can distinguish between individual substances (for example, it does not group all non-fentanyl opioids into a single category and therefore the use of this methodology would allow for the identification of specific non-fentanyl opioids, such as specific nitazenes in more recent years, as they emerge) [5, 33].

Fentanyl was the number one substance detected and contributing to AATDs in 2016 and 2017. It was also among the top substances contributing to death when detected, indicating a very high lethality. The top 12 substances contributing to more than 5% of AATDs all belong to the stimulants, opioids and alcohol classes, with cocaine, alcohol and methamphetamine specifically, being among the top 4 substances following fentanyl. These three substances seldom contributed to AATDs alone and were mainly combined with fentanyl or another opioid. Our study found that while 2 in 10 deaths caused by fentanyl were due to fentanyl alone, the vast majority of AATDs caused by fentanyl have been multidrug deaths, which is consistent with previous findings indicating that opioid related deaths are often polysubstance in nature [7, 34]. Concurrent use of opioids and stimulants is a common pattern observed among people who use drugs due to the added effects of these substances and because it balances the effects of both substance classes [35–37]. However, opioids such as fentanyl are commonly consumed unintentionally with other substances [38]. According to Health Canada’s Drug Analysis Service, which analyses seized exhibits from law enforcement agencies, the number of fentanyl identifications almost doubled between 2016 and 2017 and the vast majority of these had a co-occurring opioid or stimulant in the sample [39, 40]. A British Columbia study conducted on client provided samples at two supervised consumption sites found that samples believed to be heroin were most commonly fentanyl, seldom containing any heroin. Additionally, cocaine and methamphetamine samples were also likely to test positive for fentanyl [41]. These findings from both Health Canada and the British Columbia study indicate the presence of contaminated substances in the unregulated drug supply, which serves as one possible explanation for the common identification of AATDs caused by the combination of fentanyl and cocaine or the combination of fentanyl and methamphetamine observed in our study. While this study captures substance combinations, it does not capture the respective dose of each substance, nor does it provide any information on the clinical relevance of the substances detected for a given case or intended consumption. This observation reflects the near ubiquity of these agents and the increased risk of consuming them, especially within the landscape of contaminated drugs as seen in Canada.

Methadone, hydromorphone, oxycodone, and codeine are all non-fentanyl opioids that were among the top contributors to AATDs. These substances were most often of pharmaceutical origin, with methadone and hydromorphone being the top two substances most often acquired through a diverted prescription out of all pharmaceutical substances detected in or contributing to at least 5% of AATDs. Additionally, these non-fentanyl opioids most often contributed to death in combination with other substances, but with many different substances in small numbers (including combinations not shown in the UpSet plots).

Pharmaceutical substances contributed to death more commonly among an older demographic than non-pharmaceutical substances. Substances contributing to death were predominantly of non-pharmaceutical origin, followed by pharmaceutical origin, with combinations of pharmaceutical and non-pharmaceutical origin being the third most common origin. Thus, prescription opioid interventions should focus on both exclusive pharmaceutical use as well as concurrent use with other non-pharmaceutical substances. At the patient level, prescribers and providers should be provided with tools to test patient knowledge and educational aids they can use to raise patient awareness about the dangers and risks of using these substances alone and in combination with other substances [42–44]. At the systemic level, preventing overdoses involving these substances should focus on managing the pharmaceutical supply and risks of polypharmacy by equipping prescribers, pharmacists and patients with resources such as easy to reference information about substance risks, and how and when they are suitable for use [45–47]. However, it is important to remain mindful of possible unintended consequences of limiting access to prescriptions as this may lead patients to seek unpredictable and unsafe substances from the non-pharmaceutical drug supply and could contribute to the already increasing trend towards non-pharmaceutical deaths between 2016 and 2017 shown in this study [48–50].

In addition to opioids and stimulants, some of the substances identified as detected in or contributing to more than 5% of AATDs included benzodiazepines, acetaminophen and diphenhydramine. Diazepam and clonazepam were the top two benzodiazepines detected and contributing to AATDs in Canada in 2016 and 2017. Benzodiazepines are commonly prescribed for their function as anxiolytics, anticonvulsants, hypnotics and muscle relaxants [51]. While these substances alone were not commonly found to lead to acute toxicity deaths in this study, their compounding effect when taken with opioids increases the risk of overdose and complicates overdose reversal as naloxone (a medication that reverses an opioid overdose) does not counteract the sedative effects of benzodiazepines [52, 53]. Acetaminophen was detected in 9% of AATDs and contributed to AATDs in 1% of cases. Within the 1% of all AATDs where acetaminophen contributed to death, it was the sole contributor among 55% of deaths. Acetaminophen is among the top detected substances as it is (1) a commonly prescribed drug in combination with codeine (e.g. Tylenol#3); (2) a cutting agent used to prepare non-pharmaceutical opioids, especially heroin, as an inexpensive bulking agent, but is only present in trace amounts; and (3) is a widely available over-the counter medication (alone and in combination with other substances, including codeine) that can easily be used in a manner other than as directed by health professionals for its pain relieving properties [54–56]. Diphenhydramine is another over the counter medication identified among the top substances involved in AATDs that is also commonly used in a manner other than as directed by a health professional for its sedative effects [57]. Given that these substances are commonly used medications, their detection should be interpreted with caution as their detection does not directly imply that these substances are clinically relevant to a given AATD. These findings also highlight the need to expand AATD surveillance activities beyond the most common substances or substance classes and the usefulness of toxicology data in understanding the interplay of substances involved in death.

The majority of AATDs involved more than one substance, which is consistent with previous studies [5, 6, 8]. 70% of all AATDs had multiple substances contributing to death and 6% of AATDs in 2016 and 3% of AATDs in 2017 had no specific substances identified as contributing to death in the cause of death statement. At the substance specific level, most of the top combinations observed are either combinations classified as “dangerous” or combinations that should only be taken with “caution” according to Tripsit [29]. The high number of combinations contributing to AATDs that are classified at the “caution” risk level highlights the importance of re-evaluating the risk level of combinations and making the risks of the additive effects of polysubstance use available to the public. At the time of this study, Tripsit is one of the few public sources characterising the risks of substance use combinations [29]. Tripsit caveats that their Guide to Drug Combinations is a quick reference only and that people must always do additional research about drug combinations they intend to consume [29]. However, many people consuming non-pharmaceutical drugs may be unknowingly exposed to combinations of substances they did not intend to consume, especially given the rise of counterfeit drugs in Canada and the emergence of highly potent synthetic opioids in the drug supply, such as fentanyl, which are often mixed with other substances, such as heroin, without the user's knowledge [34, 38, 58, 59]. The synergistic effects of multiple substances add further complexity to the determination of lethality and the role of each substance as a cause of death, especially among new and emerging combinations of public health interest. The use of multiple substances poses challenges for treatment as many studies show that secondary drug use can compromise the effects of drug treatment and has been associated with poorer treatment engagement and increased fatal and non-fatal toxicity [53, 58]. Multidrug overdoses may also involve substances that lack reversal agents and yield naloxone ineffective, making it more difficult to treat overdose events [60–62].

Time trends show that new, lethal substances such as carfentanil can emerge quickly in the illegal drug supply and contribute to a large number of AATDs. Ensuring prescribed alternatives (safer supply) of substances is one way to tackle this issue as these non-pharmaceutical, highly lethal substances are introduced through illegal drug supplies and the number of AATDs with a non-pharmaceutical substance contributing to death almost doubled between 2016 and 2017. Studies looking at the impact of prescribed alternatives have found that it can reduce overdose related mortality, possibly by decreasing reliance on non-pharmaceutical supplies [63, 64]. Time trends also show that substances contributing to death change over time; diacetylmorphine (heroin) contributed to a fewer proportion of AATDs in each quarter over the two-year period, which is consistent with reports that, starting in 2013, it was slowly being replaced by fentanyl in the North American drug supply [65, 66]. Further studies looking at drug supply trends, cause of death trends and substance-related policies can help understand how each of these play a role impacting AATD trends over time.

Differences in patterns between males and females indicate the need for sex specific targeted policies, interventions and prevention strategies. For example, our findings indicate that the proportion of males represented among non-pharmaceutical AATDs is higher than the proportion of males represented among pharmaceutical AATDs, indicating the need for targeted prevention strategies focusing on non-pharmaceutical substance use among males.

Regional differences also exist; while multiple substances are detected in similar proportions across Canada, the proportions of deaths with multiple substances contributing to AATDs in each region or province varies significantly. One possible explanation for this regional variation may be differences in coroner and medical examiner practices. Coroners and medical examiners use investigative judgment, which involves subjective interpretation, to determine which substances contributed to a death. Additionally, provinces and territories have varying standard practices for selecting which substances specifically contribute to death [67]. Regional differences may also exist due to variations in the types of substances available in different provinces and territories. Health Canada’s Drug Analysis Service data on samples submitted by law enforcement agencies from various provinces shows that the types and proportions of substances available vary across provinces and territories [40]. Our results emphasize the importance of opioids, alcohol, and stimulants in multidrug AATDs, and their equal prominence in single-agent AATDs suggest that these agents are not only common but clinically relevant.

Understanding patterns and combinations of the substances and substance classes involved in AATDs can play a vital role in improving prevention and treatment interventions, overdose reversal strategies and health promotion programs. Coroners and medical examiners have previously commented on the fact that there is no common approach to the problem of medicolegal death investigation in this country, and no relevant national standards in this area [67].The practical importance of this diversity is that each coroner and medical examiner office has its own approach to toxicology testing, its own toxicology laboratory partner, and its own approach to weighing the anatomic, toxicologic, and circumstantial data that compose a final certification of death. For example, differences in toxicology testing practices likely lead to the underreporting of cannabis detected as it is a widely consumed substance not associated with a significant risk of death and therefore may not be included in all toxicology panels as testing practices vary across jurisdictions. The fact that these data originate in these provincial and territorial agencies is an important strength and an important limitation of this study. The compilation of these data in one national dataset has contributed to the development of common approaches to death investigations through the Chief Coroners, Chief Medical Examiners, and Public Health Collaborative [68]. The Collaborative is working towards more comparable and timely national data for public health surveillance and research. Continued cross-sector collaboration with coroner and medical examiner offices is needed to support consistency and better standardisation of mortality data for surveillance of and research about substance-related toxicity deaths and other public health concerns [68].

### Limitations

The results of this study should be considered in light of the following limitations. Data for this study were obtained from coroner and medical examiner files across Canada, which are collected differently across provinces and territories and not all variables collected were available in every file. Therefore, all substances involved in AATDs may not have been identified in this study and our results only show minimum estimates of prevalence. The percent of substances contributing to death when detected will be 100% for people who died in British Columbia due to differences in how the electronic data were available, and this will increase the overall proportion for the study. Only electronically available data were collected for a subset of Quebec cases, and less information may have been captured on postal code and variables used in this study that were linked to postal code (community of residence type and neighbourhood income quintiles after-tax) than was available in the physical files. British Columbia data and a subset of Ontario data were collected from electronically available files, which lacked complete substance origin and source data. Therefore, the overall involvement of pharmaceutical and non-pharmaceutical substances at the national level may be an underestimate.

Although all toxicology data were recorded, differences in what substances were tested for, testing methodologies, and laboratory equipment across and within provinces and territories – and changes in these over time – could result in inaccurate estimates in the prevalence of substances detected and contributing to death during this period. Metabolism can also affect the detection of substances (e.g., morphine and heroin). Additionally, interactions of substances, source of drug, mode of use, as well as each individual’s health history, prescribed use and dose tolerance all need to be considered when identifying what contributed to death [21].These factors could result in i) a potential bias in findings due to systematic differences in what data are available and ii) our study underestimating prevalence.

Attribution of substances to death may also vary between provinces and territories due to differences in coroner and medical examiner practices across Canada. When interpreting findings on multiple drugs, it is important to remember that there is no way of determining whether substances were intentionally consumed concurrently as prescribed or for psychoactive effects, or intentionally or unintentionally added by the drug provider. It is difficult to interpret the role of detected substances without knowing consumption patterns as substances may have been taken unintentionally, intentionally as prescribed, or intentionally for psychoactive effects.

## Conclusions

Our data show that there are important differences between substances involved in AATDs across groups of people and geographic areas as well as an increasing trend in AATDs. This study highlights the value of reporting and analyzing specific substances and including additional substance classes in substance-related toxicity death data to enhance surveillance and research. Additionally, it highlights the need to continuously track and identify new substances and combinations that contribute to accidental acute toxicity deaths (AATDs) over time. Understanding these lethal combinations can help inform the development of policies and prevention targets. The most important outcome of our study, although it may also be the most obvious is that the AATD problem is dynamic, our efforts at surveillance will need to be ongoing and subject to continuous evaluation and improvement. Occasional snapshots of the problem are not sufficient; program and policy decisions would be better informed with a more comprehensive and ongoing overdose death surveillance program that can quickly and continuously identify new substances as they emerge.

## Supplementary Information

Additional file 1. Substance class categories.

Additional file 2. Source and origin algorithms.

Additional file 3. Substance list.

Additional file 4. Sub-national substance combinations.

## Data Availability

The results, data, and figures in this manuscript have not been published elsewhere. Raw data used for this study were obtained through data sharing agreements with Canadian provinces and territories and do not belong to the authors and therefore cannot be shared publicly. However, data used in this study can be requested directly from provincial and territorial chief coroner and chief medical examiner offices.

## Abbreviations

AATDs: Accidental substance-related acute toxicity deaths
DAs: Dissemination areas
CAs: Census agglomerations
CMAs: Census metropolitan areas
NA: Not applicable
Sup: Suppressed
THC: Tetrahydrocannabinol
