# Supplementary material for: Substances and substance combinations among accidental substance-related acute toxicity deaths (AATDs) in Canada from 2016 to 2017
Source: BMC Public Health. 2025 Dec 3;26:90. doi: 10.1186/s12889-025-22777-2 (PMC12781315; doi:10.1186/s12889-025-22777-2)
Supplement: Supplementary file 1 — Additional file 1. Substance class categories. [file 12889_2025_22777_MOESM1_ESM.pdf]

Additional file 1. Substance class categories

The following table displays each substance included in the substance class categories used for our study of people who died due to acute toxicity in Canada in 2016 and 2017.

Substance type	Substances
Alcohol	Ethanol, isopropanol, methanol
Acetaminophen	Acetaminophen
Antipsychotics	Aripiprazole, asenapine, chlorpromazine, clozapine, flupenthixol, fluphenazine, haloperidol, levomepromazine, loxapine, lurasidone, olanzapine, paliperidone, pimozide, prochlorperazine, quetiapine, risperidone, ziprasidone, zuclopenthixol
Benzodiazepines	Alprazolam, bromazepam, chlordiazepoxide, clobazam, clonazepam, diazepam, etizolam, flubromazolam, flurazepam, lorazepam, midazolam, nitrazepam, oxazepam, temazepam, triazolam, unspecified benzodiazepines
Cannabinoids	Nabilone, tetrahydrocannabinol (THC)
Ethanolamine antihistamines	Diphenhydramine, doxylamine, orphenadrine
Fentanyl opioids	3-methylfentanyl, 4-fluorobutyrfentanyl, 4-fluoroisobutyryl fentanyl, acetylfentanyl, acrylfentanyl, butyrylfentanyl, carfentanil, cyclopropyl/crotonyl fentanyl, despropionyl-fentanyl, fentanyl, furanylfentanyl, methoxyacetylfentanyl, remifentanil, sufentanil
Non-fentanyl opioids	Buprenorphine, codeine, dextrorphan, diacetylmorphine (heroin), dihydrocodeine, embutramide, hydrocodone, hydromorphone, meperidine, methadone, mitragynine, morphine, oxycodone, oxymorphone, pentazocine, propoxyphene, tapentadol, thebaine, tramadol, U-47700, U-49900, U-51754
Unspecified opioids	People who died with opioids described as detected or a cause of death in the coroner or medical examiner file but no specific substances were listed.

Substance type	Substances
Gabapentinoids	Gabapentin, pregabalin
Hallucinogens	25I-NBOMe, 3-methoxyphencyclidine (3-Meo-PCP), ibogaine, lysergic acid diethylamide (LSD), mescaline, phencyclidine, psilocybin, unspecified hallucinogens
Stimulants	1,3-trifluoromethylphenylpiperazine (TFMPP), 2,5-dimethoxy-4-bromo-amphetamine, 3-fluorophenmetrazine, 4-fluoroamphetamine, alpha-pyrrolidinovalerophenone, aminorex, amphetamine, beta-phenethylamine, butylone, caffeine, cocaine, dextroamphetamine, ethylone, ethylphenidate, lisdexamfetamine, methamphetamine, methylenedioxyamphetamine (MDA), methylenedioxymethamphetamine (MDMA), methylphenidate, N-benzylpiperazine (BZP), nicotine, paramethoxyamphetamine (PMA), paramethoxymethamphetamine (PMMA), phentermine, theobromine, unspecified stimulants
Insulin	Insulin
Tri-cyclic antidepressants	Amitriptyline, amoxapine, clomipramine, desipramine, doxepin, imipramine, nortriptyline, trimipramine, unspecified tricyclic antidepressants
Other antidepressants	Bupropion, citalopram, desvenlafaxine, duloxetine, fluoxetine, fluvoxamine, mirtazapine, moclobemide, paroxetine, sertraline, trazodone, venlafaxine, vortioxetine
Z-drugs	Zolpidem, zopiclone
Other substance types	1,1-difluoroethane, 2,4-dinitrophenol, abacavir, acebutolol, acetone, acetylsalicylic acid, aconite, acyclovir, amantadine, amiodarone, amlodipine, amobarbital, anastrozole, apixaban, atenolol, atomoxetine, atorvastatin, atropine, baclofen, barbiturates, benzene, benzocaine, benztropine, benzydamine, bisoprolol, brompheniramine, bupivacaine, buspirone, butalbital, butane, capsaicin, carbamazepine, carbon monoxide, carvedilol, celecoxib, cetirizine, chloral hydrate,

Substance type	Substances
	chlorophenirамine, chloroquine, clonidine (p-hydroxyclonidine), colchicine, compressed air, creatinine, cyanide, cyclobenzaprine (N-desmethylcyclobenzaprine), cyproheptadine, dextromethorphan, diclofenac, dicyclomine, diethylene glycol, digoxin, diltiazem, dobutamine, domperidone, donepezil, efavirenz, enalapril, ether, ethylbenzene, ethylene glycol, etomidate, fenofibrate, flecainide, fluconazole, formic acid, furosemide, gammahydroxybutyrate (GHB), gliclazide, glyburide, dimenhydrinate, guaifenesin, heparin, hydrochlorothiazide, hydroxychloroquine, hydroxyzine, ibuprofen, indomethacin, irbesartan, iron, isobutane, ketamine, ketorolac, labetalol, lacosamide, lamotrigine, lansoprazole, laudanosine, levamisole, levetiracetam, levothyroxine, lidocaine, lisinopril, lithium, loperamide, losartan, meloxicam, memantine, mepivicaine, metformin, methocarbamol, methotrexate, metoclopramide, metoprolol, metronidazole, nadolol, naloxone, naltrexone, naproxen, nifedipine, nonsteroidal anti-inflammatory drugs (NSAIDs), noscapine, omeprazole, ondansetron, oxomemazine, oxybutynin, pantoprazole, pentobarbital, perindopril, phenacetin, pheniramine, phenobarbital, phenylephrine, phenytoin, piperazine, primidone, procyclidine, promethazine, propafenone, propofol, propranolol, propylene glycol, pseudoephedrine/ephedrine, quinapril, quinidine, quinine, rabeprazole, ramipril, ranitidine, rivaroxaban, rocuronium, ropinirole, rosuvastatin, salbutamol, scopolamine, secobarbital, selegiline (desmethylselegiline), sildenafil, sitagliptin, solifenacin, sulfonamides, sumatriptan, tadalafil, tamoxifen, telmisartan, terazosin, terbinafine, testosterone, theophylline, timolol, toluene, topiramate, trihexyphenidyl, trimethoprim, triprolidine, valproic acid, valsartan, varenicline, verapamil, W-18, warfarin, xylazine, xylene
Unknown substance type	People who died with no substances listed as either causing death or detected in their coroner or medical examiner files.

Note: As alcohol may be detected due to either consumption or post-mortem endogenous ethanol production, findings should be interpreted with caution.
