# Supplementary material for: Substances and substance combinations among accidental substance-related acute toxicity deaths (AATDs) in Canada from 2016 to 2017
Source: BMC Public Health. 2025 Dec 3;26:90. doi: 10.1186/s12889-025-22777-2 (PMC12781315; doi:10.1186/s12889-025-22777-2)
Supplement: Supplementary file 2 — Additional file 2. Source and origin algorithms. [file 12889_2025_22777_MOESM2_ESM.pdf]

Additional file 2. Source and origin algorithms

The opioid-related algorithms used here were developed by members of the Public Health Agency of Canada's Substance-Related Overdose and Mortality Surveillance Technical Subgroup and further adapted for the purposes of this study.

Origin allocation	Criteria for allocation
**CODEINE**	
Non-pharmaceutical origin	Without or unknown evidence of a pharmaceutical formulation or a prescription for codeine and heroin.<br>**AND**<br>With heroin* detected in toxicology.
	Without or unknown evidence of a pharmaceutical origin for codeine and heroin.<br>**AND**<br>Without heroin*, but with morphine (without evidence of a pharmaceutical origin) detected in toxicology<br>**AND**<br>History of heroin use
Pharmaceutical origin	With evidence of pharmaceutical formulation, prescription, or treatment with pharmaceutical heroin.
	With or without evidence of pharmaceutical formulation or a prescription for codeine.<br>**AND**

Origin allocation	Criteria for allocation
	With or without morphine detected in toxicology (without evidence of a pharmaceutical origin)<br>**AND**<br>Without heroin* detected in toxicology.<br>**AND**<br>Without evidence suggesting heroin use (i.e., no non-pharmaceutical opioids detected on toxicology **and** no history of heroin use)
**MORPHINE**	
Non-pharmaceutical origin	Without or unknown evidence of pharmaceutical heroin, codeine, and morphine.<br>**AND**<br>With heroin* detected in toxicology.
	Without or unknown evidence of pharmaceutical heroin, codeine, and morphine.<br>**AND**<br>With evidence suggesting heroin use (i.e. non-pharmaceutical opioids detected on toxicology or a history of heroin use)
Pharmaceutical origin	With evidence of a pharmaceutical formulation or a prescription for codeine, morphine, or heroin.<br>**AND**<br>Without heroin* detected in toxicology.

Origin allocation	Criteria for allocation
Pharmaceutical origin and non-pharmaceutical origin	With evidence of a pharmaceutical formulation or a prescription for codeine or heroin.<br>**AND**<br>With heroin* detected in toxicology.
**HEROIN**	
Pharmaceutical origin	With evidence of treatment with pharmaceutical heroin.<br>**AND**<br>Without either ATM4G (or thebaine), noscapine, papaverine, or codeine (without a prescription) detected in toxicology.<br>**AND**<br>Without or unknown evidence suggesting heroin use (i.e. no non-pharmaceutical opioids detected on toxicology **and** no history of heroin use).
Pharmaceutical origin and non-pharmaceutical origin	With evidence of treatment with pharmaceutical heroin.<br>**AND**<br>With either ATM4G (or thebaine), noscapine, papaverine, or codeine (without a prescription) detected in toxicology.
	With evidence of treatment with pharmaceutical heroin.<br>**AND**

Origin allocation	Criteria for allocation
	With evidence suggesting heroin use (i.e. non-pharmaceutical opioids detected on toxicology or a history of heroin use)
Non-pharmaceutical origin	All unknown origin responses that have not been allocated to pharmaceutical origin based on the algorithms above were moved to non-pharmaceutical origin.
**FENTANYL**	
Pharmaceutical origin	With evidence of patch or prescription
Non-pharmaceutical origin	No evidence of patch or prescription
**AMPHETAMINE**	
Non-pharmaceutical origin	Without evidence of prescription or pharmaceutical formulation of amphetamine (e.g. Adderall or Vyvanse).<br>**AND**<br>With methamphetamine detected in toxicology or indication of methamphetamine as cause of death.
Pharmaceutical origin	With evidence of prescription or pharmaceutical formulation of amphetamine (e.g. Adderall or Vyvanse).<br>**AND**<br>Without methamphetamine detected in toxicology or indication of methamphetamine as cause of death.<br>**AND**<br>Without evidence suggesting methamphetamine use (i.e, history of methamphetamine use).
Pharmaceutical origin and non-pharmaceutical origin	With evidence of methamphetamine detected in toxicology or indication of methamphetamine as cause of death.

Origin allocation	Criteria for allocation
	AND<br>With evidence of prescription or pharmaceutical formulation of amphetamine (e.g. Adderall or Vyvanse).
**KETAMINE**	
Non-pharmaceutical origin	Without evidence of treatment with ketamine.<br>**Note**: Variables documenting first aid interventions were cross-referenced for all cases involving ketamine of unknown origin for any evidence of potential ketamine use in a health care setting. In the absence of this evidence, ketamine was assigned a non-pharmaceutical origin.
**METHYLPHENIDATE**	
Pharmaceutical origin	Reviewed individual cases with methylphenidate to investigate potential origin. This included reviewing any documented mental health history for mention of attention-deficit/hyperactivity disorder and record of drug paraphernalia.
**THC**	
Non-pharmaceutical origin	Without evidence of a prescribed cannabinoid product

*Evidence of heroin may be indicated by the presence of diacetylmorphine, 6-MAM, ATM4G, thebaine, noscapine, or papaverine detected in toxicology. In the absence of any evidence of pharmaceutical heroin, these were considered to be evidence of non-pharmaceutical heroin.
