# Supplementary material for: Substances and substance combinations among accidental substance-related acute toxicity deaths (AATDs) in Canada from 2016 to 2017
Source: BMC Public Health. 2025 Dec 3;26:90. doi: 10.1186/s12889-025-22777-2 (PMC12781315; doi:10.1186/s12889-025-22777-2)
Supplement: Supplementary file 3 — Additional file 3. Substance list. [file 12889_2025_22777_MOESM3_ESM.pdf]

Additional file 3. Substance list

The following list displays all substances contributing to substance-related accidental acute toxicity deaths in Canada from 2016 to 2017.

1. 1,1- Dimethyoroethane
2. 2,5-Dimethyoxy-4-Bromoamphetamine
3. 25I-NBOMe
4. 3-Fluorophenmetrazine
5. 3-Methylfentanyl
6. 4-Fluorobutyrfentanyl
7. 4-Fluoroisobutyryl fentanyl
8. Acetaminophen
9. Acetone
10. Acetylfentanyl
11. Acetylsalicylic Acid
12. Aconite
13. Acrylfentanyl
14. Alpha-Pyrrolidinopentiophenone
15. Alprazolam
16. Amiodarone
17. Amitriptyline
18. Amlodipine
19. Amphetamine
20. Aripiprazole
21. Baclofen
22. Benztropine
23. Bromazepam
24. Buprenorphine
25. Buproprion
26. Butalbital
27. Butane
28. Butylone
29. Butyrylfentanyl
30. Caffeine
31. Carbamazepine
32. Carfentanil
33. Chlordiazepoxide
34. Chloropheniramine
35. Chloroquine
36. Chlorpromazine
37. Citalopram/escitalopram
38. Clobazam
39. Clomipramine
40. Clonazepam
41. Clonidine
42. Clozapine
43. Cocaine
44. Codeine
45. Cyclobenzaprine
46. Cyclopropyl/crotonyl fentanyl

47. Despropionyl-fentanyl
48. Desvenlafaxine
49. Dextromethorphan
50. Dextrorphan/levorphanol
51. Diacetylmorphine (heroin)
52. Diazepam
53. Digoxin
54. Dihydrocodeine
55. Diltiazem
56. Dimenhydrinate
57. Diphenhydramine
58. Doxepin
59. Doxylamine
60. Duloxetine
61. Ethanol
62. Ether
63. Ethylone
64. Ethylphenidate
65. Etizolam
66. Fentanyl
67. Flecainide
68. Flubromazolam
69. Fluoxetine
70. Flupenthixol
71. Fluvoxamine
72. Furanylfentanyl
73. Gabapentin
74. Gammahydroxybutyrate (GHB)
75. Glyburide
76. Guaifenesin
77. Haloperidol
78. Heparin
79. Hydrocodone
80. Hydromorphone
81. Hydroxychloroquine
82. Hydroxyzine
83. Ibogaine
84. Insulin
85. Isopropanol (isopropyl alcohol)
86. Ketamine
87. Labetalol
88. Lamotrigine
89. Levetiracetam
90. Levomepromazine
91. Lidocaine
92. Loperamide
93. Lorazepam
94. Loxapine
95. Lysergic acid diethylamide (LSD)
96. Meperidine
97. Metformin
98. Methadone
99. Methamphetamine
100. Methanol [Formaldehyde, Formate]
101. Methotrexate
102. Methoxyacetylfentanyl

103. Methylenedioxyamphetamine (MDA)
104. Methylenedioxymethamphetamine (MDMA)
105. Methylphenidate
106. Metoclopramide
107. Metoprolol
108. Mirtazapine
109. Mitragynine
110. Morphine
111. Naproxen
112. Nitrazepam
113. Nortriptyline
114. NSAID
115. Olanzapine
116. Oxazepam
117. Oxycodone
118. Oxymorphone
119. Paliperidone (9-hydroxyrisperidone)
120. Paroxetine
121. Phenacetin
122. Phencyclidine (PCP)
123. Pheniramine
124. Phenobarbital
125. Phenytoin
126. Pregablin
127. Pseudoephedrine/ephedrine
128. Psilocybin [Psilocyn]
129. Quetiapine
130. Quinidine
131. Risperidone
132. Rocuronium
133. Sertraline
134. Sufentanil
135. Tapentadol
136. Temazepam
137. Tetrahydrocannabinol (THC)
138. Toluene
139. Topiramate
140. Tramadol
141. Trazodone
142. Trimipramine
143. U-47700
144. Unspecified benzodiazepine
145. Unspecified hallucinogen
146. Unspecified opioid
147. Unspecified stimulant
148. Valproic acid
149. Venlafaxine
150. Verapamil
151. W-18
152. Warfarin
153. Xylazine
154. Zolpidem
155. Zopiclone
156. Zuclopenthixol
