# Supplementary material for: Substances and substance combinations among accidental substance-related acute toxicity deaths (AATDs) in Canada from 2016 to 2017
Source: BMC Public Health. 2025 Dec 3;26:90. doi: 10.1186/s12889-025-22777-2 (PMC12781315; doi:10.1186/s12889-025-22777-2)
Supplement: Supplementary file 4 — Additional file 4. Sub-national substance combinations. [file 12889_2025_22777_MOESM4_ESM.pdf]

Additional file 4. Sub-national substance combinations

The following UpSet plots display the 20 most commonly observed substances or substance combinations contributing to death among people who died due to acute toxicity in Canada in 2016 and 2017 at the provincial and regional level. Data for the Northern region, which includes Nunavut, Yukon and the Northwest Territories, are not displayed due to small cell counts.

Figure 1. Most commonly observed substances or substance combinations contributing to death among people who died accidentally due to acute toxicity in British Columbia, Canada in 2016 and 2017.

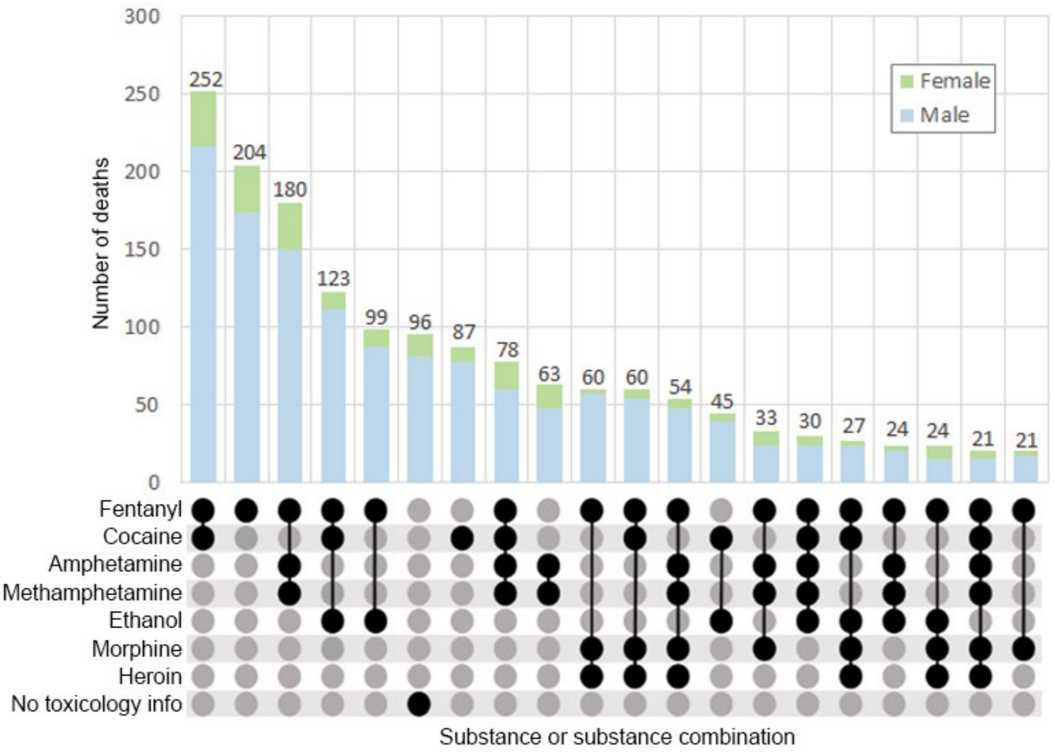

Notes: Data from British Columbia were only available for people who experienced accidental acute toxicity deaths involving unregulated drugs and/or drugs sold illicitly. As such, data for people who experienced acute toxicity deaths due solely to prescribed substances or alcohol were not available. Substances in each plot are listed in order of overall frequency, with the most frequent substance appearing at the top and the least frequent substance appearing

at the bottom. Counts are randomly rounded to base three. Cells with counts less than 10 are suppressed to protect privacy. Amphetamine is a metabolite of methamphetamine and morphine is a metabolite of diacetylmorphine (heroin). Their presence in toxicology testing could indicate that either they or their parent substance had been consumed.

Figure 2. Most commonly observed substances or substance combinations contributing to death among people who died accidentally due to acute toxicity in Alberta, Canada in 2016 and 2017.

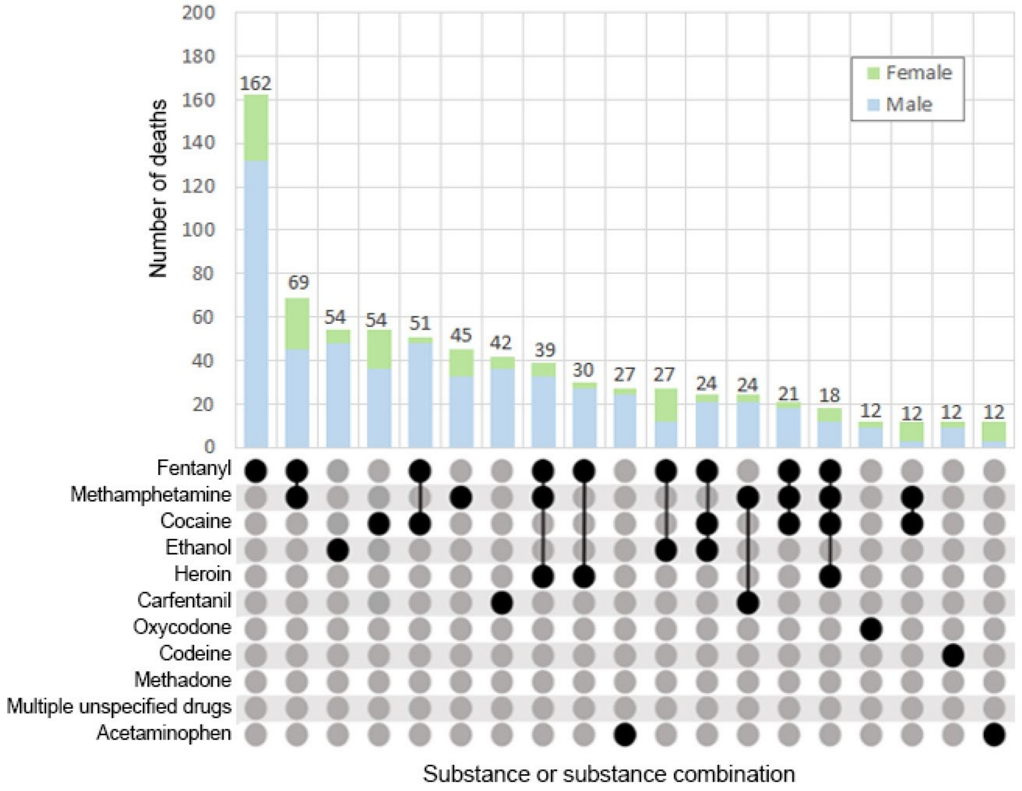

Notes: Substances in each plot are listed in order of overall frequency, with the most frequent substance appearing at the top and the least frequent substance appearing at the bottom. Counts are randomly rounded to base three. Cells with counts less than 10 are suppressed to protect privacy. Amphetamine is a metabolite of methamphetamine and morphine is a metabolite of diacetylmorphine (heroin). Their presence in toxicology testing could indicate that either they or their parent substance had been consumed.

Figure 3. Most commonly observed substances or substance combinations contributing to death among people who died accidentally due to acute toxicity in the Prairie Region, Canada in 2016 and 2017.

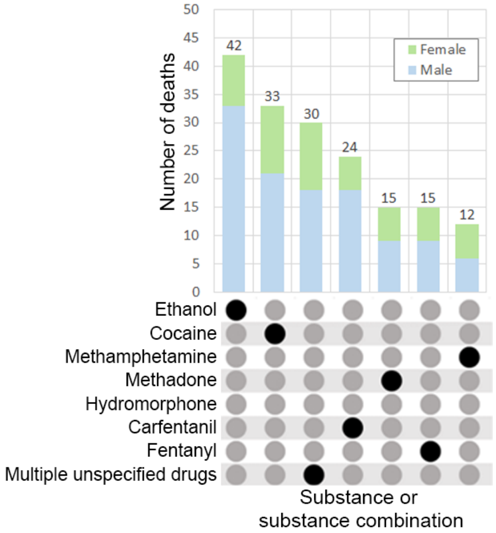

Notes: The Prairie region includes Saskatchewan and Manitoba. Substances in each plot are listed in order of overall frequency, with the most frequent substance appearing at the top and the least frequent substance appearing at the bottom. Counts are randomly rounded to base three. Cells with counts less than 10 are suppressed to protect privacy. Amphetamine is a metabolite of methamphetamine and morphine is a metabolite of diacetylmorphine (heroin). Their presence in toxicology testing could indicate that either they or their parent substance had been consumed.

Figure 4. Most commonly observed substances or substance combinations contributing to death among people who died accidentally due to acute toxicity in Ontario, Canada in 2016 and 2017.

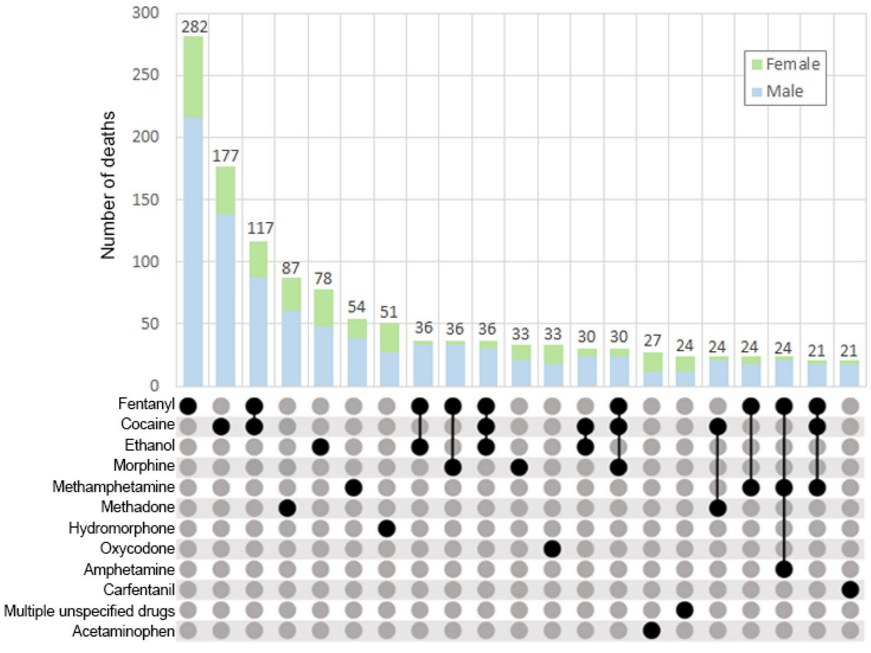

Notes: Substances in each plot are listed in order of overall frequency, with the most frequent substance appearing at the top and the least frequent substance appearing at the bottom. Counts are randomly rounded to base three. Cells with counts less than 10 are suppressed to protect privacy. Amphetamine is a metabolite of methamphetamine and morphine is a metabolite of diacetylmorphine (heroin). Their presence in toxicology testing could indicate that either they or their parent substance had been consumed.

Figure 5. Most commonly observed substances or substance combinations contributing to death among people who died accidentally due to acute toxicity in Quebec, Canada in 2016 and 2017.

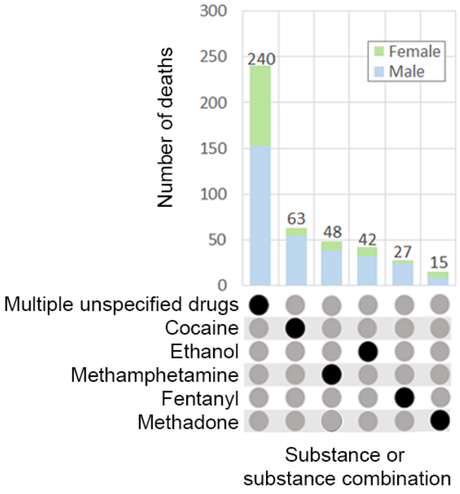

Notes: Substances in each plot are listed in order of overall frequency, with the most frequent substance appearing at the top and the least frequent substance appearing at the bottom. Counts are randomly rounded to base three. Cells with counts less than 10 are suppressed to protect privacy. Amphetamine is a metabolite of methamphetamine and morphine is a metabolite of diacetylmorphine (heroin). Their presence in toxicology testing could indicate that either they or their parent substance had been consumed.

Figure 6. Most commonly observed substances or substance combinations contributing to death among people who died accidentally due to acute toxicity in Canada's Atlantic region in 2016 and 2017.

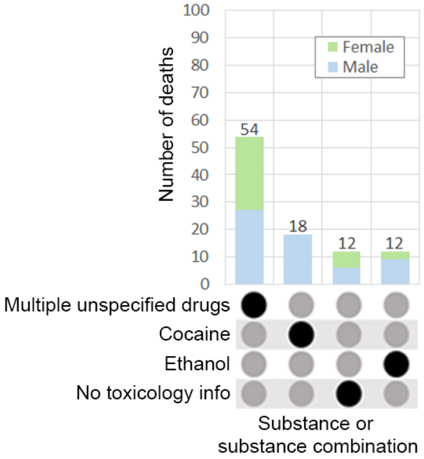

Notes: The Atlantic region includes Nova Scotia, Newfoundland and Labrador, Prince Edward Island and New Brunswick. Substances in each plot are listed in order of overall frequency, with the most frequent substance appearing at the top and the least frequent substance appearing at the bottom. Counts are randomly rounded to base three. Cells with counts less than 10 are suppressed to protect privacy. Amphetamine is a metabolite of methamphetamine and morphine is a metabolite of diacetylmorphine (heroin). Their presence in toxicology testing could indicate that either they or their parent substance had been consumed.
